# A new high-performance heterologous fungal expression system based on regulatory elements from the *Aspergillus terreus* terrein gene cluster

**DOI:** 10.3389/fmicb.2015.00184

**Published:** 2015-03-16

**Authors:** Markus Gressler, Peter Hortschansky, Elena Geib, Matthias Brock

**Affiliations:** ^1^Microbial Biochemistry and Physiology, Leibniz Institute for Natural Product Research and Infection Biology, Hans Knoell InstituteJena, Germany; ^2^Molecular and Applied Microbiology, Leibniz Institute for Natural Product Research and Infection Biology, Hans Knoell InstituteJena, Germany; ^3^Institute for Microbiology, Friedrich Schiller UniversityJena, Germany

**Keywords:** *Aspergillus niger*, secondary metabolites, transcription factor, DNA-binding motif, reporter strains, thioesterase domain

## Abstract

Recently, the *Aspergillus terreus* terrein gene cluster was identified and selected for development of a new heterologous expression system. The cluster encodes the specific transcription factor TerR that is indispensable for terrein cluster induction. To identify TerR binding sites, different recombinant versions of the TerR DNA-binding domain were analyzed for specific motif recognition. The high affinity consensus motif TCGGHHWYHCGGH was identified from genes required for terrein production and binding site mutations confirmed their essential contribution to gene expression in *A. terreus*. A combination of TerR with its *terA* target promoter was tested as recombinant expression system in the heterologous host *Aspergillus niger*. TerR mediated target promoter activation was directly dependent on its transcription level. Therefore, *terR* was expressed under control of the regulatable amylase promoter P*amyB* and the resulting activation of the *terA* target promoter was compared with activation levels obtained from direct expression of reporters from the strong *gpdA* control promoter. Here, the coupled system outcompeted the direct expression system. When the coupled system was used for heterologous polyketide synthase expression high metabolite levels were produced. Additionally, expression of the *Aspergillus nidulans* polyketide synthase gene *orsA* revealed lecanoric acid rather than orsellinic acid as major polyketide synthase product. Domain swapping experiments assigned this depside formation from orsellinic acid to the OrsA thioesterase domain. These experiments confirm the suitability of the expression system especially for high-level metabolite production in heterologous hosts.

## Introduction

*Aspergillus terreus* is a filamentous ascomycete of biotechnological and medical importance, since it produces the primary metabolite itaconic acid (Calam et al., 1939; Klement and Buchs, 2013) and the HMG-CoA reductase inhibitor lovastatin (Alberts et al., 1980; Hutchinson et al., 2000). Besides that, *A. terreus* can cause life-threatening invasive aspergillosis in immunocompromised patients (Slesiona et al., 2012b), which makes its use in biotechnological applications limited. In previous analyses we searched for secondary metabolite gene clusters that are involved in pigment formation of *A. terreus* conidia (Zaehle et al., 2014). This was of interest, since preliminary analyses suggested that the pigment in *A. terreus* differs from that found in other related *Aspergillus* species (Slesiona et al., 2012a). Coincidentally, we identified the gene cluster producing the metabolite terrein (Zaehle et al., 2014). Terrein is a metabolite with various biological activities, but its phytotoxic potential appears to be at least one of its natural functions and may increase competitiveness of *A. terreus* in the environment (Zaehle et al., 2014).

**Figure 1:**
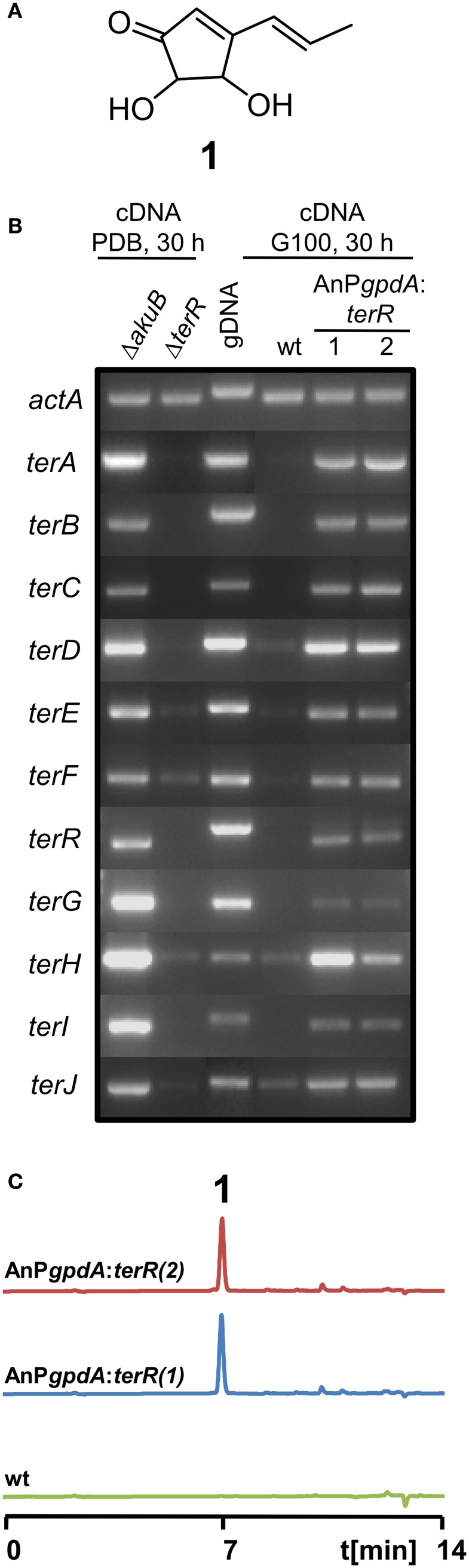
**Analysis of terrein cluster induction in dependence of *terR* expression. (A)** Chemical structure of terrein (1). **(B)** Semiquantitative RT-PCR on terrein cluster genes (*terA*—*terJ*) normalized against *actA* transcript levels. Under naturally inducing conditions (PDB) all genes from the cluster are expressed in *A. terreus* SBUG844Δ*akuB* (parental strain for gene deletions), but not in a Δ*terR* mutant. The wild-type SBUG844 shows no cluster expression under non-inducing conditions (G100), whereas strains constitutively expressing *terR* under the *A. nidulans gpdA* promoter (AnP*gpdA*:*terR* No. 1 and 2) induce the cluster. **(C)** HPLC analysis of G100 culture extracts from wild type (wt) and *terR* overexpressing strains AnP*gpdA*:*terR* No. 1 and 2. The metabolite peak for terrein is denoted by “1.”

Interestingly, terrein (Figure 1A) is produced in large quantities, since short-term cultivation in simple potato dextrose broth resulted in more than 1 g of terrein per liter (Zaehle et al., 2014) and even higher yields have been described for cultivation under more optimized conditions (Xu et al., 2012; Yin et al., 2012). This implies that genes from the terrein gene cluster may be expressed at very high levels.

The terrein cluster contains seven genes responsible for terrein biosynthesis (*terA-F*, *terR*), whereby *terA* encodes a non-reducing polyketide synthase (PKS) that produces polyketides of different chain length namely 2,3-dehydro-6-hydroxymellein (C10), orsellinic acid (C8), and 4-hydroxy-6-methylpyrone (C6). The subsequent formation of the cyclopentenoic structure of terrein is proposed to derive from an oxidative ring contraction of the isocoumarinic precursor 2,3-dehydro-6-hydroxymellein. While the terrein gene cluster contains further co-regulated genes, these genes might be dispensible (*terH*, *terI*), because deletion only reduces the final terrein production level or are involved in metabolite export (*terG*, *terJ*) (Zaehle et al., 2014). Importantly, the gene cluster encodes its own transcriptional activator at locus tag ATEG_00139, which is called TerR. TerR is a transcriptional activator with a GAL4-type Zn_2_Cys_6_ zinc binuclear cluster DNA-binding domain. This type of DNA-binding domain is very common in fungi and is the most common type of DNA-binding domains in transcriptional regulators of secondary metabolite gene clusters from filamentous fungi such as GliZ for gliotoxin biosynthesis from *Aspergillus fumigatus* (Bok et al., 2006), AflR for aflatoxin biosynthesis from *Aspergillus nidulans* and *Aspergillus flavus* (Yu et al., 1996) or ApdR for aspyridone biosynthesis from *A. nidulans* (Bergmann et al., 2007). A genomic deletion of *terR* resulted in complete loss of terrein production, suggesting that this transcription factor essentially contributes to cluster expression (Zaehle et al., 2014). However, neither the DNA binding sites recognized by TerR, nor the signals leading to TerR activation have been identified so far. However, if TerR is sufficient to drive expression of cluster genes, we assumed that a combination of TerR together with promoters from its cluster could enable the development of a new heterologous expression system.

Several filamentous fungi have been used for the recombinant production of proteins, among them especially fungi of industrial importance with GRAS status (generally regarded as safe) such as *Aspergillus niger*, *Aspergillus oryzae*, *Trichoderma reesei*, *Acremonium chrysogenum*, and *Penicillium chrysogenum* (Sharma et al., 2009). Several post-transcriptional bottlenecks have been reported to limit the production of proteins. These may consist of incorrect protein folding and subsequent degradation, low secretion efficiency, extracellular degradation or hyperglycosylation and several attempts have been made to overcome these limitations (Ward, 2012). However, filamentous fungi have not only been used for protein production, but are also used for the production of lignofuels or metabolic intermediates such as citric acid and the production of secondary metabolites such as antibiotics and other therapeutically useful compounds (Lubertozzi and Keasling, 2009). Despite specific limitations in all expression systems including the codon-adaptation of the target gene and RNA stability, the initial high-level expression of a target gene is the first key step for high production rates.

In general, a strong promoter is used to drive gene expression. In this respect, expression systems frequently rely on endogenous promoters from primary metabolism that are either constitutively active or can be regulated by applying specific inducing or repressing conditions. Examples are alcohol and aldehyde dehydrogenase (*alcA*; *aldA*), glucoamylase (*glaA*), (Taka) amylase (*amyA*; *amyB*), glyceraldehyde-3-phosphate dehydrogenase (*gpdA*), sucrase (*sucA*), acetamidase (*amdS*), endoxylanase (*exlA*), superoxide dismutase (*sodA*), or cellobiohydrolase I (*cbhI*) (Sharma et al., 2009; Fleissner and Dersch, 2010). Additionally, multiple integrations of an expression construct frequently increases the overall transcription of the target gene, but a linear increase is mainly limited to the first 5–6 copies (Verdoes et al., 1993). Due to these limitations, an inducible system with a promoter that is strongly activated and produces high transcript levels already in single-copy is generally favored.

Interestingly, although high secondary metabolite production rates have been described for several fungal species, information on the use of the involved regulatory elements to drive recombinant gene expression is limited. To analyze, whether elements from the *A. terreus* terrein cluster might be suitable for such a new recombinant expression system, we first analyzed, whether TerR is sufficient to drive terrein cluster expression and identified the respective DNA binding sites. With this knowledge, elements from the cluster were tested by reporter gene expression for their performance in the heterologous host *A. niger*. Finally, the expression system was used to heterologously produce secondary metabolites from *A. terreus* and *A. nidulans* in *A. niger*.

## Materials and methods

### Strains and culture conditions

Strains used in this study are summarized in Table S1. For maintenance and during transformation procedures, all *Aspergillus* strains (*A. nidulans* wild type FGSC A4, *A. terreus* SBUG844 and its derivatives and all *A. niger* FGSC A1144 derivatives) were cultivated for 4 days at 37 or 30°C on solid *Aspergillus* minimal media (AMM) containing 2% agar (http://www.fgsc.net/methods/anidmed.html). When required, either hygromycin B (140 μg/ml, Carl Roth GmbH; Germany), pyrithiamine hydrobromide (0.1 μg/ml) or phleomycin (80 μg/ml) (both Sigma Aldrich; Germany) were added. Conidia were harvested in sterile water from solid media and filtered over 40 μm cell strainers (VWR; Germany). Liquid cultures were generally used in a 50 ml scale and were inoculated with 1 × 10^6^ conidia per ml. Cultures were incubated at 30°C for 48–72 h as specified in the respective experiments. The following liquid media were used: AMM with 100 mM glucose (AMM-G100) or 1% casamino acids (AMM-CA1%), potato dextrose broth (PDB, Sigma Aldrich), and yeast/malt extract medium (YM, 5 g/l peptone, 3 g/l yeast extract, 3 g/l malt extract).

### Bacterial expression and purification of TerR polypeptides for SPR analysis

All oligonucleotides used in this study are listed in Table S2. The gene sequences encoding for TerR residues 1–153 (TerR_1−153_), 43–138 (TerR_43−138_), and 35–138 (TerR_35−138_) were amplified by PCR from cDNA of *A. terreus* wild-type strain SBUG844 that was cultivated for 48 h at 30°C on PDB medium. The following oligonucleotides introducing 5′-*Nde*I and 3′-*Hind*III restriction sites were used: P1/2 for TerR_1−153_, P3/4 for TerR_43−138_, and P5/4 for TerR_35−138_. The fragments were cloned into the pET-29a vector (Novagen; Germany). TerR polypeptides were produced by autoinduction in *E. coli* Rosetta2 (DE3) cells grown at 26°C in 1·l Overnight Express Instant TB Medium (Novagen) in the presence of 1 mM Zn(OAc)_2_. Fifteen to twenty grams wet cells were collected by centrifugation, resuspended in 200 ml lysis buffer (20 mM HEPES, 150 mM NaCl, 10 μM Zn(OAc)_2_, 5 mM β-Mercaptoethanol, 1 mM AEBSF, pH 7.5) and disrupted using an Emulsiflex C5 high pressure homogenizer (Avestin; Germany). Cleared cellular extracts were loaded on a SP Sepharose HP (GE Healthcare; Germany) column and eluted with a salt gradient up to 1 M NaCl. Pooled fractions containing TerR_1−153_, TerR_43−138_, or TerR_35−138_ were adjusted to 150 mM NaCl and applied on a Cellufine Sulfate (Millipore; Germany) column that was equilibrated with 20 mM HEPES, 150 mM NaCl, 10 μM Zn(OAc)_2_, 5 mM β-Mercaptoethanol, pH 7.5, followed by elution with a gradient to 1 M NaCl. Peak fractions were concentrated with an Amicon Ultra-15 10K centrifugal filter device and purified to homogeneity by size exclusion chromatography on a Superdex 75 prep grade column (GE Healthcare) by using 20 mM HEPES, 150 mM NaCl, 10 μM Zn(OAc)_2_, pH 7.5 as running buffer. TerR proteins were stored in 50% v/v glycerol at −20°C. The absolute molecular mass of TerR_35−138_ was determined by static light scattering experiments on a miniDawn TREOS monitor in series with an Optilab T-rEX differential refractometer (Wyatt Technology Europe; Germany). TerR_35−138_ was chromatographed on a Superdex 200 10/300 GL column (GE Healthcare) and molar mass was calculated using ASTRA 6 software (Wyatt Technology Europe).

### Surface plasmon resonance measurements

Real-time analyses were performed on a Biacore 2000 system (GE Healthcare) at 25°C. DNA duplexes were produced by annealing complementary 18 bp oligonucleotides using a 5-fold molar excess of the non-biotinylated oligonucleotide. The dsDNA was injected on flow cells of a streptavidin (Sigma Aldrich)-coated CM3 sensor chip at a flow rate of 10 μl/min until the calculated amount of DNA that gives a maximum TerR binding capacity of 50 RU had been bound. TerR proteins were injected in running buffer (10 mM HEPES pH 7.4, containing 150 mM NaCl, 0.005% (v/v) surfactant P20, 5 mM β-Mercaptoethanol and 1 μM ZnCl_2_) at concentrations from 12.5 to 6400 nM. Sample injection and dissociation times were set to 60 and 120 s at a flow rate of 30 μl/min. Refractive index errors due to bulk solvent effects were corrected with responses from DNA-free flow cell 1 as well as subtracting blank injections. Kinetic raw data were processed and globally fitted with Scrubber 2.0c (BioLogic Software) using a 1:1 interaction model including a mass transport term.

### Genetic manipulation of *A. niger* and *A. terreus*

All strains generated in this study are listed in Table S1. The number of genomic integrations of all constructs generated in this study was determined by Southern blot analyses with digoxygenin labeled probes. Blots were developed by chemoluminescence imaging using CDP-star as recommended by the manufacturer (Roche; Germany). For details on specific strain constructions refer to supplementary experimental procedures. In brief, all *A. terreus terR* expression constructs were amplified from genomic DNA of *A. terreus* SBUG844. The constructs either contained the native *terR* promoter or the *terR* promoter was replaced by the *A. nidulans gpdA* or the *A. oryzae amyB* promoter. The *terR* terminator sequence was maintained in all constructs. Either the pyrithiamine (*ptrA*) or hygromycin B (*hph*) resistance cassette was used as a selectable marker in transformations of *A. terreus* SBUG844, *A. niger* FGSC A1144, or *A. niger* FGSC A1144_P*terA*:*lacZ*. Transformations were performed as previously described (Zaehle et al., 2014). For β-galactosidase producing reporter strains the *lacZ* gene from *E. coli* was fused with different promoters. The following promoters were used: The *A. nidulans gpdA* promoter, the *A. terreus terA* promoter in unidirectional (P*terA*) and bidirectional (P*terA*/*B*) orientation, the *A. terreus terC* promoter and the mutant versions P*terC*m1 and P*terC*m2, in which the putative TerR binding site “BS4” was exchanged or mutated. Fusion constructs were cloned into plasmids containing either the *ptrA* or *hph* resistance cassette and used for transformation *A. terreus* SBUG844, *A. niger* FGSC A1144, or *A. niger* FGSC A1144_P*amyB*:*terR* (P2 strain). For analysis of the bidirectional *terA*/*B* promoter a second reporter gene was required and a synthetic codon-optimized *tdTomato* gene coding for a red fluorescent protein was selected (gene accession KP100262). The *lacZ* and *tdTomato* genes were fused in both orientations with P*terA*/*B* and cloned in plasmids containing the *hph* resistance cassette. These reporter plasmids were used for transformation of *A. niger* FGSC A1144 or *A. niger* FGSC A1144_P*amyB*:*terR* (P2 strain).

### Expression of *terA* and *orsA* in *A. niger* P2 strain and construction of the expression plasmid SM-Xpress

For expression of the *A. terreus terA* gene and the *A. nidulans orsA* gene in the *A. niger* P2 strain (containing the P*amyB*:*terR* construct), the *terA* promotor was either fused to the *terA* or *orsA* gene, whereas the respective natural terminator sequences were maintained. The fusion constructs were ligated into an *hph* containing vector and used for transformation of the *A. niger* P2 strain. To ease heterologous expression of polyketide synthases in the *A. niger* P2 strain the expression plasmid SM-Xpress was constructed. A plasmid containing the phleomycin (*ble)* resistance cassette (AnP*gpdA*:*ble*:*trpC*^T^) was linearized with *EcoR*I. A 786 bp fragment of the *A. terreus terA* promoter was amplified with oligonucleotides P6/7 and fused with a 363 bp *trpC* terminator (*trpC*^T^) from *A. terreus* (P8/9) and cloned into the *EcoR*V site of the linearized vector by *in vitro* recombination using the InFusion HD Cloning Kit (Clonetech laboratories; Germany). Thus, the resulting plasmid “SM-Xpress” contains a phleomycin resistance cassette as selection marker and the fusion of P*terA* and *trpC*^T^, which are separated by a *Nco*I site. This vector was used for domain swapping experiments of the *A. nidulans orsA* gene, in which the TE domain of OrsA was replaced by the TE domain from TerA. The *orsA* gene except its TE domain and the TE domain from *terA* were PCR amplified and fused with the *Nco*I restricted plasmid SM-Xpress *via in vitro* recombination. Plasmids were used for transformation of FGSC A1144_P*amyB*:*terR* (P2).

### Metabolite extraction

To analyze cultures for secondary metabolite production, culture broth was extracted with an equal volume of ethylacetate and the procedure was repeated once. Both fractions were combined and evaporated under reduced pressure. Evaporated residues were solved in 1 ml methanol and filtered. Standard metabolite analyses were performed on an Agilent 1100 series HPLC-DAD system coupled with a MSD trap (Agilent Technologies; Germany) operating in alternating ionization mode as previously described (Gressler et al., 2011).

### Quantification of orsellinic acid

To quantify metabolite production levels from the direct and the coupled expression system orsellinic acid from the TerA PKS was selected. Metabolites were analyzed on an analytical Shimadzu HPLC system equipped with a DAD type SPD-M20A using a Zorbas Eclipse XDB C8 column (4.5 × 150 mm; 5 μm) with H_2_O + 0.1% formic acid (buffer A) and methanol (buffer B) as solvents. The following gradient was applied: 0–0.5 min = 10% B; 0.5–12 min from 10 to 90% B; 12–14 min from 90 to 100% B; 14–17 min = 100% B; 17–18 min from 100 to 10% B; 18–21 min = 10% B. A standard curve was generated using defined concentrations of purified orsellinic acid (range from 3.9 to 250 μg/ml). Cultures were cultivated for 48 h at 30°C and 200 rpm in AMM-G100+Gln50 liquid medium. Mycelium was collected for dry weight determination and culture supernatants were extracted twice with ethylacetate. Extracts were evaporated, solved in 1 ml methanol and filtered. Different dilutions from each sample in a total volume of 10 μl were loaded to the columns. Peak areas for orsellinic acid at 254 nm were quantified and production levels were calculated against the dry weight biomass.

### RNA isolation, cDNA synthesis and semiquantitative PCR

*A. terreus* SBUG844 and *A. niger* FGSC A1144 strains were cultivated for 30 or 48 h in PDB, AMM-G100, YM and AMM-CA1% media. Mycelia were ground under liquid nitrogen and RNA was isolated by the RiboPure RNA purification kit (Thermo Scientific; Germany). Residual genomic DNA was removed by the DNA-free kit (Thermo Scientific) and cDNA was synthesized by the Revert Aid Reverse Transcriptase (Thermo Scientific) using anchored oligo dT primers. Semiquantitative PCR was performed as described (Zaehle et al., 2014) previously using oligonucleotides P10–P35. Transcripts were normalized against the actin gene (*actA*, ATEG_06973) for *A. terreus* strains and against the glycerinaldehyde-3-phosphate dehydrogenase gene (*gpdA*, est_fge1_pm_C_70216) for *A. niger* strains.

### Determination of β-galactosidase activity and tdTomato fluorescence intensity

β-Galactosidase activity was determined as previously described (Gressler et al., 2011). In brief, mycelia from *A. niger* strains containing *lacZ* fusion constructs were harvested from cultures grown for 48 h at 30°C in PDB, AMM, YM, or AMM-CA1% media. Mycelia were ground under liquid nitrogen and resuspended in 50 mM MOPS buffer (pH 7.5) with 2 mM MgCl_2_ and 10 mM β-mercaptoethanol. After centrifugation at 21,000 × *g* supernatants were used for determination of β-galactosidase activity using *o*-nitrophenyl-β-galactoside as substrate (Gressler et al., 2011). To detect the fluorescence intensity of *A. niger* transformants containing *tdTomato* fusion constructs, cell-free extracts were prepared as described above. After determination of protein concentrations by the Bradford assay (BioRad; Germany) protein concentrations were adjusted to 2.0, 1.0, and 0.5 mg/ml. 200 μl of the dilutions were transferred to a black flat-bottom 96 well plate (Nunc; Germany) and analyzed in a microplate reader (FLUOstar Omega, BMG Labtech; Germany). Plates were shaken for 1 min in double orbital mode and emission was detected at 590 nm using an excitation wavelength of 544 nm (50 flashes/well, top reading, gain 2000). The parental *A. niger* strains A1144 and P2 that contained no *tdTomato* gene served as negative controls and were subtracted from the fluorescence values of reporter strains. Specific activities were expressed as fluorescence units per mg protein. To calculate relative expression levels, fluorescence intensities (FI) of reporter strains were normalized against fluorescence intensities of an *A. niger* strain expressing the *tdTomato* gene under control of the *A. nidulans gpdA* promoter using the following formula: Relative fluorescence intensity (rFI) [%] = (FI_reporter strain_ − FI_P2_)/(FI_AnP*gpdA*_ − FI_WT_). All assays were performed in biological and technical triplicates from at least three individual transformants.

### Accession numbers

The DDBJ/EMBL/GenBank nucleotide sequence database accession number for the *Aspergillus* codon-optimized *tdTomato* gene reported in this paper is KP100262.

## Results and discussion

### The transcription factor TerR is essential for expression of terrein cluster genes

Previously, we have shown that *A. terreus* produces large quantities of the metabolite terrein (Figure 1A) and we discovered that the responsible gene cluster contains its own transcription factor TerR, which is encoded at locus tag ATEG_00139 (Zaehle et al., 2014). Gene deletion analysis revealed that TerR is indispensable for terrein production in *A. terreus*, since *terR* deletion resulted in complete loss of terrein production during cultivation on inducing potato dextrose broth (PDB) (Zaehle et al., 2014). To confirm that TerR is directly involved in the activation of the gene cluster, comparative semiquantitative RT-PCR analyses between *A. terreus* wild type and *terR* mutant (Δ*terR*) were performed. Under inducing conditions, all genes from the cluster spanning the region from *terA* – *terJ* (locus tags ATEG_00145—ATEG_00135) were actively transcribed in the wild type (Figure 1B). While all analyzed genes were strictly dependent on TerR for induction, *terE*, *F* and *H* showed some background expression in the Δ*terR* background. Thus, TerR seems to act as a transcriptional activator with special importance for transcription of the key polyketide synthase TerA, which shares a bi-directional promoter with the *terB* gene. Interestingly, among the 11 genes spanning the cluster, six of them (*terA*/*B*; *terE*/*F*, and t*erH*/*I*) share bi-directional promoters, implying that either multiple putative TerR binding sites are found in these promoters or that single binding sites can activate expression in both directions. However, the control of gene expression from bi-directional promoters is frequently found in fungal secondary metabolite gene clusters. As examples, in the aflatoxin gene cluster from *Aspergillus parasiticus* 12 out of 25 genes share a bi-directional promoter (Yu et al., 2004) and in the gliotoxin gene cluster from *Aspergillus fumigatus* (Forseth et al., 2011) 8 out of 12 genes are controlled by shared promoter sequences. Furthermore, similar to our results on TerR-dependency for terrein cluster activation, the Zn_2_Cys_6_ binuclear transcription factor *gliZ* from the gliotoxin cluster is essential for activation of cluster genes (Bok et al., 2006).

### TerR is sufficient for activation of terrein cluster genes

In *A. terreus* terrein is only produced under specific environmental conditions, suggesting that transcription and activation of *terR* is itself under the control of other transcription factors that mediate environmental signals toward terrein production. Thus, to analyze, whether TerR production under non-inducing conditions is sufficient to stimulate terrein production in *A. terreus*, we aimed in the exchange of the native *terR* promoter against the constitutively active *gpdA* promoter from *Aspergillus nidulans* (Punt et al., 1990). The resulting P*gpdA:terR* fusion construct was used to transform an *A. terreus* wild-type strain. Indeed, when transformants were grown on complete glucose minimal medium, which is a repressive condition for terrein production in *A. terreus*, semiquantitative RT-PCR revealed induction of all cluster genes and high amounts of terrein were produced (Figures 1B,C). Thus, *terR* expression in *A. terreus* wild type is strictly dependent on specific inducing signals, but once expressed, TerR is sufficient to induce all genes required for terrein production. Similarly, the transcription factors ApdR (Bergmann et al., 2007), AflR (Liu and Chu, 1998), and GliZ (Bok et al., 2006) have been shown to induce expression of the respective cluster when overproduced in the homologous host. Therefore, our data indicate that promoter elements of the terrein cluster contain specific DNA binding sites that are recognized by TerR.

### The TerR zinc binuclear cluster DNA-binding domain has a monomeric structure

**Figure 2:**
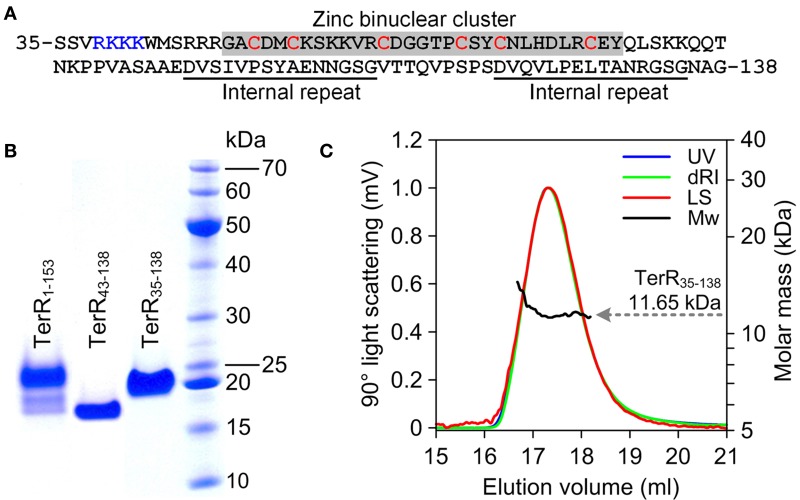
**Characterization of recombinant TerR polypeptides used in this study. (A)** Amino acid sequence of TerR_35-138_ used for SPR interaction analysis with specific DNA fragments. The *N*-terminal basic stretch at positions 38 to 47 and the zinc cluster region are highlighted. **(B)** SDS-PAGE analysis of purified TerR fragments as indicated at the specific lanes. **(C)** Analysis of oligomeric state of TerR_35-138_ in solution as determined *via* size exclusion chromatography and multiangle static light scattering. The light scattering signal (LS) is shown overlaid with the calculated molar mass (Mw) across the elution profile as monitored by the absorbance at 280 nm (UV) and changes of the refractive index (dRI).

Since TerR was able to induce all genes required for terrein synthesis, we expected that conserved DNA binding sites are present in the promoter regions of genes spanning the terrein cluster. TerR belongs to the family of transcriptional activators with a GAL4-type Zn_2_Cys_6_ zinc binuclear cluster DNA-binding domain. This type of transcription factors is one of the most abundant transcriptional regulators present in fungi and involved in the regulation of primary and secondary metabolic processes (MacPherson et al., 2006). Their DNA-binding domain is generally located at the *N*-terminus of the protein and is sufficient to recognize its target sequence (Todd et al., 1998). Additionally, most GAL4-type transcription factors contain a coiled-coil domain near the *N*-terminus that is involved in dimerization and results in the recognition of consensus repeats (Fitzgerald et al., 2006). Interestingly, no coiled-coil domain is detected in the *N*-terminus of TerR, instead RADAR (Rapid Automatic Detection and Alignment of Repeats in protein sequences) analysis (Heger and Holm, 2000) detected an unusual amino acid repeat at positions 97–111 and 121–135 with unknown function. Thus, for analysis of the TerR subunit composition and identification of DNA-binding sites we first selected an *N*-terminal peptide of 153 amino acids (TerR_1−153_) that was cloned into an expression vector with or without *N*-terminal His-tag. Unfortunately, regardless the peptide version (tagged or untagged) and the purification procedure, we were unable to obtain a monodispersed protein fraction and MALDI-TOF MS/MS analysis showed co-purification of *N*- and *C*-terminal degradation products (Figure 2B). A similar phenomenon was observed during purification of the transcriptional activator AlcR of the ethanol utilization pathway in *A. nidulans* (Felenbok et al., 1988; Cahuzac et al., 2001). An AlcR_1−197_ fragment enclosing the DNA-binding domain could not be purified to homogeneity (Lenouvel et al., 1997). Therefore, we truncated the TerR domain to a TerR_35−138_ fragment (Figure 2A). This protein fragment was purified to >98% homogeneity as judged by SDS-gel electrophoresis (Figure 2B) and analytical size exclusion chromatography. Multiangel static light scattering analysis revealed a molar mass of 11.65 kDa (Figure 2C), demonstrating that the purified protein exclusively consisted of monomers in solution (theoretical molar mass of 11.35 kDa). Thus, besides the transcriptional activator AlcR, TerR seems to form another example of GAL4-type transcription factors with monomeric solution structure.

### TerR recognizes CGG direct repeat consensus sequences in the terrein cluster

**Figure 3:**
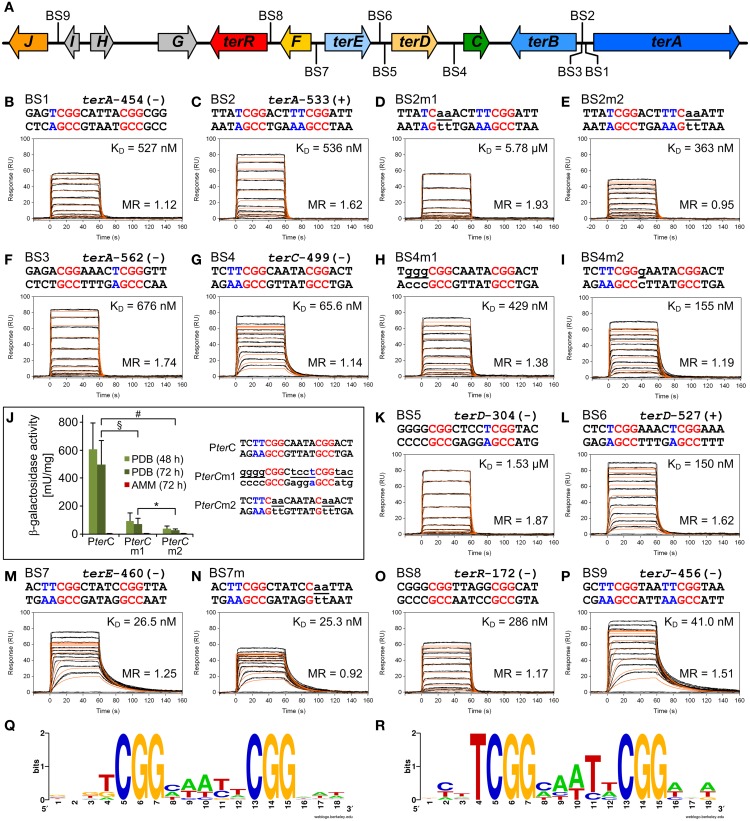
***In vitro* TerR binding to a consensus motif identified in promoters of the *A. terreus* terrein biosynthesis gene cluster and *in vivo* verification of *in vitro* data. (A)** Schematic presentation of the terrein gene cluster. Intergenic positions of the consensus TerR-binding motif identified by the SCOPE motif finder suite **(Q)** are annotated with BS1–9. **(B–I,K–P)** Real-time *in vitro* SPR interaction analysis of TerR_35-138_ with DNA containing the predicted natural or mutated (denoted by the letter “m”) binding sites from promoters of terrein cluster genes. Sequences of DNA duplexes used for SPR analysis are shown on top of the sensorgrams. Numbers represent the CGG direct repeat motif positions relative to the start of the open reading frame. CGG half-sites and 5′-flanking thymidines are highlighted in red and blue. Substituted nucleotides relative to the wild-type sequence are underlined and shown in lowercase letters. TerR_35-138_ binding responses from triplicate injections of different concentrations (black lines) are overlaid with the best fit derived from a 1:1 interaction model including a mass transport term (red lines). Dissociation constants (K_D_) are plotted inside the sensorgrams. For details on the specific binding motifs refer to the main text. **(J)** *In vivo* verification of the P*terC* binding motif BS4. P*terC:lacZ* reporter strains were grown for 48 and 72 h on PDB or for 72 h on AMM and β-galactosidase activity was determined. Highest activity is observed with the native *terC* promoter (P*ter*C), whereas activity is significantly reduced by replacement of the native binding site (BS4) against the low affinity BS5 from the *terD* promoter (P*terC*m1) or when the two CGG half-sites of BS4 are replaced by CAA triplets (P*terC*m2). No activity for any reporter is detected on AMM. Statistical significance (p < 0.01) was calculated by the student's unpaired *t*-test using data from at least three independent transformants measured in biological triplicates. Error bars represent SEM. ^§^P*terC* vs. P*terC*m1; ^#^P*terC* vs. P*terC*m2; ^*^P*terC*m1 vs. P*terC*m2, all significances for PDB, but not AMM. **(R)** WebLogo sequence consensus motif of the experimentally mapped high affinity TerR binding sites BS1, BS2, BS4, BS6, BS7, and BS9.

To identify putative TerR binding sites in the terrein gene cluster an *in silico* prediction of candidates constituting regulatory DNA motifs was performed on the intergenic regions of *terA*—*terJ* (Figure 3A) using the SCOPE motif finder suite (Chakravarty et al., 2007). A SPACER bipartite motif with the direct CGG half-site repeat consensus 5′-**CGG**HHHBN**CGG**-3′ (Figure 3Q) was identified that was present in all promoter regions of the cluster, with the exception of *terG*, *terI*, and *terH*, whereby the latter genes were dispensable for terrein production (Zaehle et al., 2014). Interestingly, the intergenic region that acts as a bidirectional promoter of *terA* and *terB* contained three putative binding sites that were annotated as BS1, BS2, and BS3 (Figure 3A). To analyze recognition of these binding sites by the TerR_35−138_ fragment, protein: DNA real-time surface plasmon resonance (SPR) biosensor interaction analyses were performed. For this purpose, oligonucleotides with a four-nucleotide overhang at the 5′-CGG half-site and a three-nucleotide overhang at the 3′-CGG half-site were deduced. Biotinylated oligonucleotides were hybridized with complementary unbiotinylated anti-strands and immobilized on streptavidin-coated CM3 sensor chips to give a maximum response (R_max_) of 50 resonance units (RU) when bound by a single monomeric TerR_35−138_ domain. Kinetic SPR binding responses of TerR_35−138_ to BS1-BS3 fitted with *K*_D_-values ranging between 0.5 and 0.7 μM. Interestingly, while steady state binding analysis showed that only one monomer binds to the BS1 sequence (Figure 3B, Table 1), BS2 and BS3 revealed that a significant fraction of DNA duplexes were bound by two monomers at these binding sites (Figures 3C,F).

**Table 1:** **Dissociation constants and stoichiometry of analyzed TerR_35-138_:DNA interactions**.

**DNA duplex (Binding site)**	**Mw duplex (Da)**	**DNA bound (RU)**	R_max_ calculated^*^ (RU)	**R_max_ measured (RU)**	**molar ratio TerR:DNA**	***K_D_***
BS1	11405	52.4	52.1	58.5 ± 0.5	1.12	527 ± 3 nM
BS2	11401	51.6	51.3	82.9 ± 0.1	1.62	536 ± 3 nM
BS2m1	11399	54.7	54.5	105.2 ± 0.7	1.93	5.78 ± 0.06 μM
BS2m2	11339	51.2	51.0	48.4 ± 0.8	0.95	363 ± 2 nM
BS3	11403	52.0	51.8	90.3 ± 0.1	1.74	676 ± 3 nM
BS4	11403	55.9	55.9	63.6 ± 0.1	1.14	65.6 ± 0.6 nM
BS4m1	11404	53.1	52.8	72.8 ± 0.1	1.38	429 ± 2 nM
BS4m2	11403	52.9	52.7	62.6 ± 0.1	1.19	155 ± 1 nM
BS5	11408	52.9	52.6	98.6 ± 0.1	1.87	1.53 ± 0.06 μM
BS6	11403	52.3	52.1	84.6 ± 0.1	1.62	150 ± 1 nM
BS7	11403	50.1	49.9	62.3 ± 0.1	1.25	26.5 ± 0.3 nM
BS7m	11401	52.4	52.2	48.2 ± 0.1	0.92	25.3 ± 0.2 nM
BS8	11406	52.1	51.8	60.4 ± 0.1	1.17	286 ± 2 nM
BS9	11402	51.7	51.5	77.8 ± 0.2	1.51	41.0 ± 0.4 nM

* *R_max_ calculated = 11350 (Mw TerR_35-138_)/Mw DNA × DNA bound*.

To analyze the specificity for the predicted motif, BS2 was selected for mutations in the first and second CGG half-sites. Mutation of the 5′-CGG to CAA led to a predominant loss of TerR_35−138_ binding (Figure 3D). In contrast, mutation of the 3′-CGG to CAA altered the *K*_D_ from 536 to 363 nM, but simultaneously reduced the number of TerR_35−138_ units that bind to the sequence (Figure 3E). While 1.6 monomers bound to the native sequence, only 0.95 monomers were found to bind at the mutated sequence. A similar phenomenon was observed when the second CGG half-site was mutated in BS7 (Figures 3M,N, Table 1). This implies that the 5′-CGG half-site is required for high affinity binding, whereas the 3′-CGG half-site attracts a second monomer to the motif. To confirm this assumption and to define a consensus sequence, we tested all other binding sites that were predicted by SCOPE using SPR analysis (Figure 3). Indeed, all predicted binding sites were recognized by TerR_35−138_, whereby *K*_D_ values ranged from 26 nM to 1.5 μM with either a tendency to bind only one or two TerR_35−138_ fragments. In this respect, binding of a second monomer seems to be favored when the last nucleotide of the five base pair spacer is a thymidine (BS2, BS3, BS5, BS6, and BS9, Figure 3). In contrast, motifs, in which the spacer between the two CGG half-sites was reduced to four instead of five base pairs as well as motifs that harbor inverted CGG half-sites were not recognized by TerR (data not shown). We additionally observed that sequences that contained one or two “T” nucleotides in front of the 5′-CGG half-site had a tendency for high-affinity binding (BS1, BS2, BS4, BS6, BS7, BS9). To analyze the importance of these residues, we mutated the two “T” nucleotides in BS4 into “G” nucleotides (BS4m1; Figure 3H). Indeed, the affinity of the mutated binding site decreased by a factor of 6.5. In contrast, mutation of “C” into “G” directly behind the 5′-CGG half-site only showed a minor effect on affinity (Figure 3I).

### A basic stretch at the TerR *N*-terminus promotes high affinity DNA binding

The selected TerR_35–138_ fragment contained a basic stretch at positions 38–47 that is followed by the predicted DNA-binding domain. In this respect, TerR appears to possess similar features as the regulator of ethanol utilization AlcR from *Aspergillus nidulans*, which also recognizes DNA-binding sites in monomeric form as mentioned in Section “The TerR Zinc Binuclear Cluster DNA-Binding Domain has a Monomeric Structure.” For AlcR it has been shown that a basic sequence at the *N*-terminus is important for high affinity DNA binding, since it strengthens the interaction of the monomer with the phosphate backbone (Nikolaev et al., 1999). In order to elucidate the importance of this basic stretch in TerR, we generated a TerR_43−138_ fragment that lacked four basic amino acids present in the TerR_35−138_ fragment (Figure 2). When the TerR_43−138_ fragment was tested by SPR analysis on selected binding sites, a similar order of binding affinity was observed as for the TerR_35−138_ fragment. However, *K*_D_-values increased by a factor of 7–22, confirming that the basic stretch is essential for tight binding to the DNA phosphate backbone (Figure S2). Additionally, while some binding motifs showed the tendency to bind two TerR_35−138_ fragments, the TerR_43−138_ fragment exclusively recognized these motifs as a single monomer (Table 2). This implies that recognition of the second CGG half-site is not only dependent on the sequence motif, but also on the general affinity of the TerR fragment toward DNA binding.

**Table 2:** **Dissociation constants and stoichiometry of analyzed TerR_35-138_:DNA interactions**.

**DNA duplex (Binding site)**	**Mw duplex (Da)**	**DNA bound (RU)**	R_max_ calculated^*^ (RU)	**R_max_ measured (RU)**	**Molar ratio TerR:DNA**	***K_D_***
BS2	11401	56.3	50.5	52.4 ± 0.2	1.04	3.73 ± 0.03 μM
BS4	11403	58.5	52.4	45.9 ± 0.1	0.88	1.07 ± 0.06 μM
BS4m1	11404	57.7	51.7	49.3 ± 0.3	0.95	9.3 ± 1 μM
BS4m2	11403	58.0	52.0	45.9 ± 0.1	0.88	1.37 ± 0.05 μM
BS6	11403	57.3	51.3	43.4 ± 0.1	0.85	2.05 ± 0.09 μM
BS7	11403	60.0	53.8	48.9 ± 0.1	0.91	591 ± 2 nM
BS8	11406	60.9	54.6	58.0 ± 1.0	1.06	17.2 ± 0.5 μM
BS9	11402	59.6	53.4	49.3 ± 0.1	0.92	820 ± 4 nM

* *R_max_ calculated = 10218 (Mw TerR_43-138_)/Mw DNA × DNA bound*.

Thus, comparison of the different features of the TerR fragments and sequence analyses lead to the following conclusions: (i) A TerR_1−153_ fragment is unstable *in vitro*, but the basic stretch at the *N*-terminus is required for high affinity DNA binding. (ii) The 5′-CGG half-site is of major importance for sequence recognition and binding. (iii) High affinity binding sites are characterized by a “T” before the 5′-CGG half-site. Similarly (iv) when the last nucleotide of the spacer is a “T,” there is a tendency for a second monomer to bind to the 3′-CGG half-site, but only in the presence of the basic amino acid stretch in the TerR domain. (v) The consensus sequence for TerR high affinity binding sites is 5′-TCGGHHWYHCGG –3′ (Figure 3R).

### *in vitro* binding affinity of TerR_35−138_ is reflected by *in vivo* promoter activity

The *in vitro* analyses of the TerR_35−138_ fragment showed that BS4 reflects a high affinity binding site with a *K*_D_ of 66 nM, but tendency for single monomer binding (Figure 3G). BS4 is the only confirmed binding site in the intergenic region of *terC* (ATEG_00143, Figure 3A), which is no longer expressed when *terR* is deleted (Figure 1B). Thus, we were interested, whether the *in vitro* binding properties of the predicted binding sites reflect promoter activation *in vivo*. To address this question, we generated three reporter constructs. The first construct contained the native *terC* promoter fused with the *E. coli lacZ* gene (P*terC:lacZ*). For the second construct, BS4 from the *terC* promoter was exchanged with BS5 and also fused with the *lacZ* gene resulting in P*terC*m1*:lacZ*. BS5 is one of two binding sites in the intergenic region of *terD* (ATEG_00142), which showed a 23 times reduced TerR_35−138_ binding affinity *in vitro*, but a tendency for binding two monomers (Figure 3K). Finally, for the last construct, the two CGG half-sites of BS4 were replaced by CAA in P*terC* and fused to the *lacZ* gene resulting in P*terC*m2*:lacZ*. These mutations in both CGG half-sites were assumed to lead to a complete loss of binding site recognition. The *A. terreus* wild-type strain was transformed with the three constructs and several independent transformants with single genomic integrations were selected for analysis of β-galactosidase activity under inducing and non-inducing conditions (Figure 3J). As expected, none of the transformants revealed β-galactosidase activity when cells were grown under non-inducing conditions (AMM). In contrast, activity significantly increased, when cells were grown on PDB medium. Here, β-galactosidase activity was at least 6 times higher from strains containing the native BS4 site compared to strains containing the less affined BS5 site (P*terC*m1). Moreover, compared to the native promoter, the conversion of both CCG half-sites to CAA (P*terC*m2) reduced promoter activity by a factor 17. Thus, the binding sites predicted from *in silico* and *in vitro* analyses are indeed important for promoter activation by TerR and *in vitro* parameters reflect the *in vivo* situation.

### TerR activates the *terA* promoter in the heterologous host *A. niger*, but requires a promoter exchange

**Figure 4:**
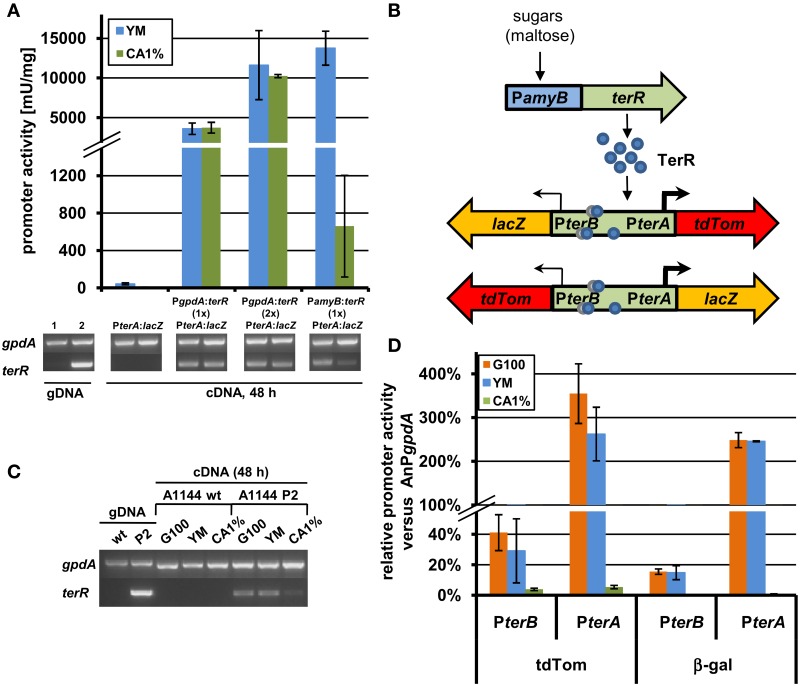
**Analysis of recombinant *terR* and *terA* expression in *A. niger* by semiquantitative RT-PCR, LacZ, and tdTomato reporter activity. (A)** Semiquantitative RT-PCR (lower panels) and β-galactosidase activity (upper panel) of *A. niger* A1144_P*terA*:*lacZ* either without *terR* or co-expressing the *terR* gene under control of the *A. nidulans gpdA* promoter (P*gpdA:terR*, P*terA:lacZ*; with one (1×) or two (2×) copies of P*gpdA:terR*) or the *A. oryzae amyB* promoter (P*amyB*:*terR*, P*terA:lacZ*). Expression of *terA* requires *terR* co-expression and is dependent on *terR* expression levels. *A. niger gpdA* was used as reference gene in semiquantitative RT-PCR analyses. Lane 1, control genomic DNA (gDNA) of *A. niger* P*terA:la*cZ; Lane 2, control gDNA of *A. niger* P*amyB*:*terR*, P*terA:la*cZ. **(B)** Scheme of the *terR* dependent expression system using the bi-directional promoter of *terA* and *terB*. After sugars induced expression of *terR*, the TerR protein (blue circles) recognizes the three bindings sites in the bi-directional *terA/B* promoter and activates the expression of reporter genes (*lacZ* and *tdTomato*) presumably in different strength (indicated by arrows). **(C)** Semiquantitative RT-PCR analysis of wild type *A. niger* A1144 and A1144_P*amyB*:*terR* (P2) expressing *terR* under control of the *amyB* promoter. *terR* is strongly expressed on P*amyB* inducing media (G100 and YM), but hardly detectable on CA1% medium. **(D)** Relative promoter activity of P*terA* and P*terB* from the reporter system described in **(C)**. The parental strain P2 and the reporter strains P2_P*terA*:*lacZ*_P*terB:tdTom* and P2_P*terB*:*lacZ*_P*terA:tdTom* were cultivated for 48 h on G100, YM and CA1% and β-galactosidase activity and fluorescence intensity from cell-free extracts were determined. All values were normalized against reporter activities from the *A. nidulans gpdA* promoter (AnP*gpdA*:*lacZ* and AnP*gpdA*:*tdTom*) that were used as 100% reference values. Each experiment was carried out with three independent mutants in biological and technical triplicates.

In order to generate a heterologous expression system using regulatory elements from the terrein gene cluster, we selected the filamentous fungus *A. niger*, which is frequently used in biotechnological applications (Sharma et al., 2009). Here, an analysis was required that tested the possibility of TerR mediated induction of terrein cluster promoters in the heterologous host. Thus, to analyze, whether TerR is sufficient to activate *terA* in *A. niger*, different β-galactosidase reporter strains were generated (Figure 4 and Figure S1). First, the promoter of *terA* (P*terA*), which contained the three DNA binding sites BS1-3 was fused with the *E. coli lacZ* gene and transferred to *A. niger*. After cultivation on different media, β-galactosidase activity hardly exceeded the background level, indicating that the *terA* promoter is not recognized by *A. niger* transcription factors that are present under the applied conditions (Figure 4A and Figure S3). Unfortunately, when *terR* under its native promoter was additionally introduced into the P*terA:lacZ* strain, again no β-galactosidase activity was detected (Figure S3). This indicated that either (i) TerR is not able to activate the *terA* promoter in the heterologous system or that (ii) the native promoter of TerR is not recognized in *A. niger*. To test these assumptions, we analyzed the expression of *terR* by semiquantitative PCR. Indeed, no *terR* transcript was detected, which confirms that specific regulatory elements from *A. terreus* are required to drive activation of *terR* expression (Figure S3). However, to show that TerR can also activate the *terA* promoter in a heterologous host, *terR* was fused with the constitutively active glyceraldehyde-3-phosphate dehydrogenase promoter (P*gpdA*) from *A. nidulans* and the resulting P*gpdA:terR* construct was introduced into the *A. niger* strain with the P*terA:lacZ* reporter. Indeed, a strong β-galactosidase activity was detected, which confirmed the specific recognition of *terA* promoter elements by TerR even in a heterologous system (Figure 4A). Additionally, β-galactosidase activity doubled in a strain that contained two copies of the P*gpdA:terR* constructs, indicating that TerR levels are the rate limiting step in *terA* promoter activation (Figure 4A). In conclusion, the native *terR* promoter is not stimulated and transcribed in *A. niger*, but when TerR is produced under control of an active promoter, this transcription factor is sufficient to stimulate expression of *terA* also in the heterologous system.

### Control of *terR* expression under the inducible *amyB* promoter allows regulated gene expression from the bi-directional *terA/B* promoter

Experiments in Section “TerR Activates the *terA* Promoter in the Heterologous Host *A. niger*, but Requires a Promoter Exchange” revealed that control of *terR* expression under the *gpdA* promoter leads to constitutive activation of the *terA* promoter. In order to regulate expression, the *amyB* promoter was selected, since it was previously shown to be highly active in the presence of maltose or glucose, but only shows low background activity on sugar-free media such as 1% casamino acids (CA medium) (Ward, 2012; Zaehle et al., 2014). Indeed when the *A. niger* strain carrying the P*terA:lacZ* reporter was transformed with a P*amyB:terR* construct, high β-galactosidase activity was obtained when cells were cultivated on maltose containing YM medium, whereas activity after cultivation in CA medium was approximately 20 times lower (Figure 4A). This was also reflected by *terR* expression levels that were highly abundant on YM medium, but hardly detected on CA medium (Figure 4A). This again confirms that high *terR* expression levels are crucial for strong activation of target promoters. This also indicates that expression from promoters of the terrein cluster can be regulated by modifying the production level of the TerR regulator.

The selected terrein cluster promoter is assumed to depict a bi-directional promoter, because it separates the reading frames of *terA* and *terB* that are transcribed in opposite directions as already described in Section “The Transcription Factor TerR is Essential for Expression of Terrein Cluster Genes.” Interestingly, it has been shown that in the bi-directional promoters of penicillin biosynthesis from *A. nidulans* (*acvA*/*ipnA*) (Brakhage, 1997) and cephalosporin biosynthesis from *Acremonium chrysogenum* (*pcbAB*/*pcbC*) (Menne et al., 1994) transcription rate is favored in one direction. To test transcriptional activation from the *terA*/*B* promoter, the *lacZ* gene and a codon-optimized tdTomato gene encoding a red fluorescent protein were selected as reporters in both reading directions (Figure 4B). An *A. niger* strain carrying a single copy of the P*amyB:terR* construct (P2 strain) that shows the expected expression pattern of *terR* under inducing and non-inducing conditions (Figure 4C), was used as recipient strain for the different reporter constructs. Four to eight single copy transformants from each construct were analyzed in a pre-screening approach to test for variations in the expression pattern due to positioning effects (Minetoki et al., 1998; Liu et al., 2003; Blumhoff et al., 2013). All transformants revealed a similar tendency of expression levels (±25% from average). Therefore, three independent transformants were selected from each construct and strains were cultivated in biological triplicates for determination of the average promoter activation rate in comparison to the *gpdA* control promoter. Selected transformants with single copy integration were grown on glucose, YM or CA medium and screened for β-galactosidase activity and fluorescence intensity from cell-free extracts. All measurements were background corrected against extracts from an *A. niger* strain without reporter integration. Reporter activities were normalized against activities obtained from expression of the respective reporters under control of the *A. nidulans gpdA* promoter (Figure 4D).

Results from both reporters show that (i) *terA*/*B* promoter activity in both directions is strongly induced on glucose and YM medium, whereas activity remained near background values when cells were grown on non-inducing CA medium. (ii) Reporter activity in direction of the *terA* gene exceeded that of the *gpdA* promoter by up to three times, whereas activity in *terB* direction only reached 20–40% of the control value. Thus, transcription in the direction of the polyketide synthase gene *terA* exceeds that of *terB* by 8–14-fold. Additionally, the relative accumulation of TdTomato was slightly higher than that of the β-galactosidase, which could be due to increased stability of the codon-optimized *TdTomato* gene. In conclusion, these results indicate that *terA*/*B* promoter activation is directly dependent on the level of the TerR regulator.

Previous approaches to develop high-level expression systems used strongly expressed and, preferentially, inducible promoter elements for direct control of target gene expression. Examples are the thiamine promoter (P*thiA*) for expression in *A. oryzae* (Shoji et al., 2005), the xylose induced *xyl1* promoter in *A. chrysogenum* (Blatzer et al., 2014), synthetic promoters containing the human estrogen receptor (hERα) response elements in *A. nidulans* and *A. niger* (Pachlinger et al., 2005) or the *Escherichia coli* tetracycline resistance operon (Tet-on system) in *A. fumigatus* and *A. niger* (Vogt et al., 2005; Meyer et al., 2011). Here, we identified that the activity of the artificial promoter that controls *terR* expression is amplified *via* TerR-dependent activation of the *terA* promoter. This assumption derives from the following observation: In previous studies we have shown that the *gpdA* and *amyB* promoter display similar activation levels on G100 medium (Zaehle et al., 2014). Thus, in case there is no signal amplification, reporter activity from the coupled system should be equivalent to P*gpdA*-driven activity. However, the analyses performed here show that reporter activity increased by a factor >2 when P*amyB* controls *terR* expression and the reporter is under *terA* control. Therefore, the coupled system of terrein cluster elements leads to an amplification of the strength of the promoter that is used to regulate *terR* expression. We assume that activity of promoters that are even stronger than P*amyB* could be amplified in our new coupled system. Although it might be expected that a saturation of the *terA* promoter may occur at a specific TerR level, this could either be compensated by increasing the number of *terR* binding sites in the *terA* promoter or by adding additional copies of the fusion of P*terA* with the gene of interest. However, a preliminary analysis of multicopy integrants with the P*terA*:*lacZ* construct revealed that expression levels run into saturation at approximately five integrations.

### The P*amyB:terR*/P*terA* system is suitable for high-level heterologous secondary metabolite production

**Figure 5:**
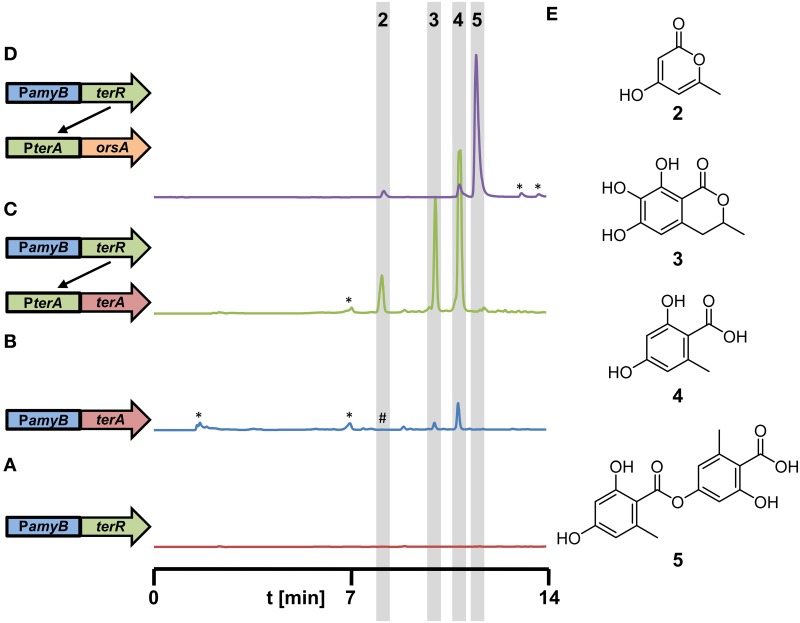
**Metabolite detection from culture filtrates of recombinant *A. niger* strains expressing different polyketide synthases under control of the *terR*/P*terA* expression system**. All strains were grown for 48 h in AMM-G100 medium with 20 mM glutamine as nitrogen source. **(A–D)** HPLC profiles from **(A)** A1144_P*amyB*:*terR* (P2, parental control strain), **(B)** A1144_P*amyB*:*ter*A (control strain) with conventional direct *terA* expression, (Zaehle et al., 2014), **(C)** P2_P*terA*:*terA* (coupled system), and **(D)** P2_P*terA*:*orsA* (coupled system). A schematic drawing of the integrated constructs is given on the left next to the respective profile. **(E)** Structures of metabolites identified in the extracts are: 4-hydroxy-6-methylpyrone (2), 6,7-dihydroxymellein (3), orsellinic acid (4), and lecanoric acid (5). ^*^Indicate non-reproducible metabolite peaks; ^#^refers to (2) that is only visualized by loading concentrated extracts of the P*amyB*:*terA* transformants.

Due to the high reporter activities obtained from the *terA* promoter in combination with the P*amyB:terR* control element, we were interested in the use of this system for the heterologous production of secondary metabolites. In a previous study, we expressed the terrein synthase gene *terA* under direct control of the *amyB* promoter in *A. niger* (Zaehle et al., 2014). Sufficient quantities of the TerA metabolites orsellinic acid (4), 6,7-dihydroxymellein (3) and 4-hydroxy-6-methylpyrone (2) were produced (for molecular structures refer to Figure 5E). However, we assumed that metabolite production in the heterologous system could strongly exceed the quantity of metabolites compared to the previous construct. To confirm this hypothesis, the P2 strain with the single copy of the P*amyB:terR* integration was transformed with the *A. terreus terA* gene under control of its native *terA* promoter. Transformants were cultivated in glucose minimal medium, which was selected since HPLC profiles of the P2 strain showed no significant background metabolite production under this condition (Figure 5A). The metabolic profile of transformants was directly compared with that of a P*amyB:terA* strain that was cultivated under the same conditions (Figures 5B,C). Indeed, although all three metabolites were detected in similar ratios as detected from the P*amyB:terA* strain, the amount of metabolites produced by the new expression system strongly exceeded that of the former strain. In order to quantify the increase of metabolite production in the coupled system, we selected three P*amyB*:*terA* single copy and three P*amyB*:*terA* double copy integrants in the *A. niger* A1144 genetic background (Zaehle et al., 2014) and four single copy and two double copy P*terA*:*terA* integrants from the P2 strain. Transformants were cultivated in AMM-G100+Gln50 for 48 h, supernatants were extracted and orsellinic acid concentration was quantified by HPLC and normalized against the mycelial dry weight (Figure S4). Although production rates varied among independent transformants, the single and double copy strains with the P*amyB*:*terA* construct produced on average 0.37 (±0.19) and 0.43 (±0.13) mg orsellinic acid per gram of dried mycelium. Single copy P*terA*:*terA* integrants produced on average 1.16 (±0.64) and double copy integrants 3.20 (±1.69) mg of orsellinic acid per gram of dried mycelium. This results in an approximated 3-fold increase in the coupled expression system for single copy and a 7.5-fold increase for the double copy integrants in comparison to the direct expression system under control of the *amyB* promoter. Although increases in production rates may be metabolite dependent, these results reflect the increased transcription rate from the coupled expression system as shown in Figure 4D and confirms the suitability of the system for heterologous expression.

In another approach, we selected the *orsA* PKS gene from *A. nidulans* for heterologous expression in *A. niger*. Previous analyses have shown that the *orsA* gene is induced during the cocultivation of *A. nidulans* with the bacterium *Streptomyces hygroscopicus* (Schroeckh et al., 2009). Structure elucidation of the metabolites produced by OrsA required a 14 liter cocultivation of both organisms to obtain sufficient amounts of metabolites. Interestingly, orsellinic acid and lecanoric acid, a depside of two orsellinic acid molecules, were identified. However, it remained unclear, which of the two products is the major metabolite produced by OrsA and whether OrsA is directly performing the transesterification of two orsellinic acid molecules (Schroeckh et al., 2009). Therefore, we generated a *PterA:orsA* construct that was transferred into the *A. niger* P2 strain and single copy integrants were tested for product formation in 50 ml culture scale. Ethylacetate extracts revealed one major and two minor metabolites (Figure 5D) that were identified by HRESI-MS as lecanoric acid (5), orsellinic acid (4) and 4-hydroxy-6-methylpyrone (2) and the identity was confirmed by reference against known standards and ^1^H and ^13^C NMR spectra of purified lecanoric acid (Figure S5 and supplementary experimental procedures). Since lecanoric acid was by far the most prominent metabolite produced by all independent transformants, it can be assumed that the thioesterase (TE)-domain of OrsA performs the transesterification of the *p*-hydroxyl-group of one orsellinic acid molecule with the carboxyl-group of a second molecule rather than releasing orsellinic acid by simple hydrolysis. A similar mechanism of depside formation from orsellinic acid-derived metabolites might be present in lichen-forming fungi in which lecanoric acid and derivatives thereof constitute major secondary metabolites (Parrot et al., 2015). However, whether the involved polyketide synthases perform depside formations has not yet been characterized in detail (Armaleo et al., 2011).

### A new expression vector enables rapid cloning and modification of secondary metabolite genes

**Figure 6:**
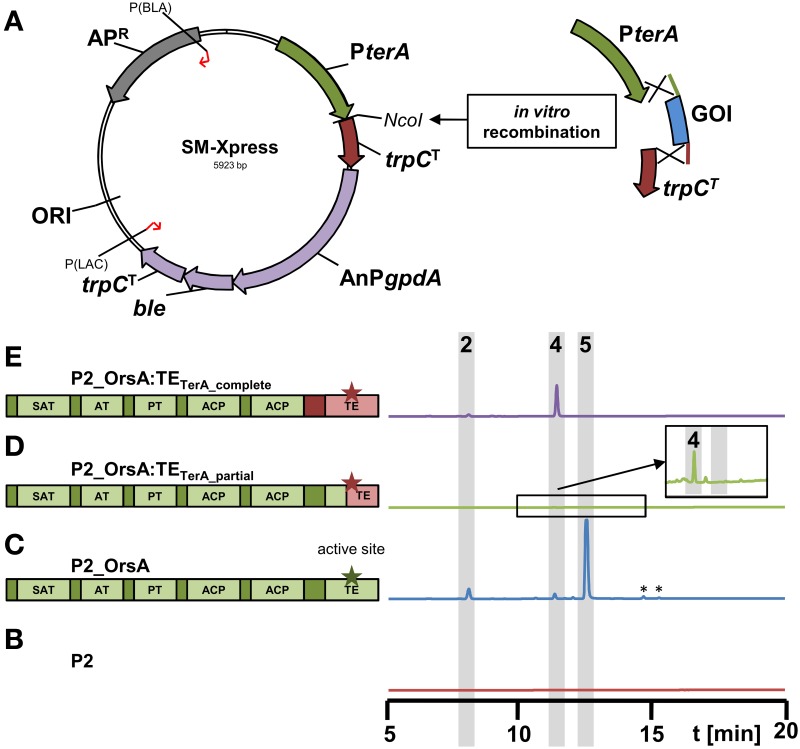
**Scheme of the new expression vector SM-Xpress and its use for rapid cloning of polyketide synthases in domain swapping experiments. (A)** The plasmid SM-Xpress contains the native *terA* promoter and a *trpC* terminator and allows cloning of a gene of interest (GOI) by restriction cloning or *in vitro* recombinantion into the *Nco*I site. Due to a phleomycin resistance cassette (AnP*gpdA:ble:trpC^T^*), the plasmid is suitable for direct transformation of the *A. niger* P2 strain expressing *terR* under P*amyB* control. **(B–E)** HPLC profiles of culture filtrates from *A. niger* P2 strains grown for 40 h in AMM-G100 + Gln50 and expressing different versions of the original *A. nidulans orsA* gene or chimeric versions with exchanged thioesterase (TE) domains as depicted in the schemes on the left. Green parts originate from *orsA* and pink parts from *A. terreus terA*. Asterisks indicate the active sites of the TE domains. **(B)** P2 control strain (P*amyB*:*terR*), **(C)** P2_A1144_P*terA*:*orsA*, **(D)** P2_P*terA:orsA:TE_terA_partial_* and **(E)** P2_P*terA:orsA:TE*_terA_complete_. Indicated peaks correspond to 4-hydroxy-6-methylpyrone (2), orsellinic acid (4) and lecanoric acid (5). Note that a partial exchange of the TE domain **(D)** leads to a chimeric protein that hardly releases products and only trace amounts of orsellinic acid are detected (magnified inlay). ^*^Indicate non-reproducible metabolite peaks.

In order to ease the use of the heterologous expression system, we constructed a plasmid that allows rapid generation of expression constructs called SM-Xpress. For this purpose, we used a plasmid with a phleomycin resistance cassette in which the *ble* gene was controlled by the *A. nidulans gpdA* promoter. Subsequently, the 786 bp *terA* promoter and a 363 bp fragment of the *A. terreus trpC* terminator were amplified by PCR and integrated into the plasmid by *in vitro* recombination. This resulted in plasmid “SM-Xpress” that contained a *Nco*I restriction site that separated promoter and terminator and allowed insertion of the gene of interest by restriction ligation or *in vitro* recombination (Figure 6A). Resulting overexpression plasmids are suitable for direct transformation of the *A. niger* P2 strain that contains the P*amyB:terR* construct.

To test this system, we went back to the *orsA* gene from *A. nidulans*. As stated in Section “The P*amyB*:*terR*/P*terA* System is Suitable for High-Level Heterologous Secondary Metabolite Production,” OrsA predominantly produced lecanoric acid and we hypothesized that the depside formation from two orsellinic acid molecules is mediated by the thioesterase domain (TE) of this enzyme. In general, TE domains are important for product release, but can also perform an interketide esterification (cross-coupling) between the carboxyl-function of the polyketide and an alcohol, whereby in a recent example of melleolide formation in *Armillaria mellea* the hydroxyl group derives from a sesquiterpene (Lackner et al., 2013). To confirm that the depside formation in lecanoric acid is specific for the OrsA TE domain, we performed an *in vitro* recombination between the *orsA* gene lacking its TE domain and the TE domain from *A. terreus terA* that only releases monomers of its products (Figures 5C,D and Zaehle et al., 2014). In this approach, two different fusions were made: (i) the complete TE domain starting directly 3′ of ACP2 was removed from *orsA* and fused with the complete TE domain of *terA* (*orsA*:TE_terA_complete_; Figure 6E). (ii) The TE domain from *orsA* was exchanged at a conserved region of the active site of the TE domain (*orsA*:TE_terA_partial_; Figure 6D). The *A. niger* P2 strain was transformed with the fusion constructs and transformants were analyzed for their metabolic profile (Figures 6B-E). The partial exchange of the active site only resulted in trace amounts of orsellinic acid (Figure 6D), indicating that this exchange led to loss of function of the TE domain. Additionally, it indicates that a functional TE domain is required for product release. This is in agreement with other investigations that showed that TE domains are required for efficient product release from PKS enzymes, whereby the specific product release mechanism is mainly directed by the TE domain (Du and Lou, 2010; Xu et al., 2013).

In contrast to the chimeric protein with partial exchange of the TE domain at the active site, *A. niger* transformants expressing the chimeric *orsA* with the complete *terA* domain exclusively produced orsellinic acid in high yields and lecanoric acid was no longer detected (Figure 6E). Due to these results we provide the first experimental evidence that the depside formation in lecanoric acid is attributed to the TE domain of *orsA*. This opens a new avenue for domain swapping experiments, in which the *orsA* TE domain could be used for fusions with other polyketide synthases to generate new depsides. Furthermore, these results not only confirm the suitability of our expression system for rapid analysis of products formed from secondary metabolite gene clusters, but also shows that the system provides a tool for rapid combinatory domain swapping experiments.

## Conclusions and outlook

In this study we confirmed specificity of the transcriptional activator TerR from the terrein gene cluster for its target promoters. Furthermore, we were able to identify DNA-binding motifs recognized by TerR. Interestingly, TerR predominantly binds as a monomer to a single CGG half-site, but a second CGG motif that is separated by a five nucleotide spacer can be bound by a second monomer. High affinity sites with tendency for binding of two monomers are characterized by the consensus motif TCGGHHWYHCGGH. At least one site with this high affinity motif is present in all promoters of genes required for terrein synthesis. Interestingly, no high-affinity consensus motifs are detected in *terG*, *terH*, and *terI* that are dispensable for terrein production, but show the same TerR dependent regulation pattern. Thus, single half-site motifs may be present in these promoters, but these are difficult to predict by bioinformational methods. On the contrary, the *terA* promoter contains three CGG direct repeat motifs and especially expression of the *terA* gene is strongly activated by TerR. The *terC* promoter contains only a single, but high affinity binding motif and its expression level is much lower than that from *terA*. Therefore, the number and probably also the distance of the motifs relative to the transcriptional start point appear important for high-level transcriptional activation. To confirm this assumption, future studies will replace all binding sites in the *terA* promoter by motifs from other promoters (such as BS6 and BS7). Furthermore, the number of binding sites will be increased to analyze the effect on the promoter activation potential.

Although there might be space for further optimization of the system, heterologous expression analyses in *A. niger* clearly showed that a combination of TerR with its *terA* target promoter is highly efficient and leads to an amplification of the expression level of the promoter that controls *terR* expression. Thus, additional promoters will be used to control *terR* expression to analyze the maximum amplification rates that can be obtained. Another important aspect is the transfer of the system to other fungi of industrial importance. While preliminary analyses showed that the system also works in other *Aspergillus* species, we will study the functionality of the system in yeasts such as *Saccharomyces cerevisiae* and various Basidiomycetes that are amenable to genetic modifications.

Last but not least, our proof of principle studies for heterologous expression of secondary metabolite genes by the TerR/P*terA* coupled system demonstrate an easy and rapid method for production of secondary metabolites. The vector SM-Xpress allows fast cloning of genes and eases domain swapping experiments. By this approach, we were able to show that the TE domain of OrsA performs a depside formation and future studies will use this domain for generating chimera with other polyketide synthases.

### Conflict of interest statement

The authors declare that the research was conducted in the absence of any commercial or financial relationships that could be construed as a potential conflict of interest.

## References

[B1] Alberts A. W. Chen J. Kuron G. Hunt V. Huff J. Hoffman C.. (1980). Mevinolin: a highly potent competitive inhibitor of hydroxymethylglutaryl-coenzyme A reductase and a cholesterol-lowering agent. Proc. Natl. Acad. Sci. U.S.A. 77, 3957–3961. 10.1073/pnas.77.7.3957 6933445 PMC349746

[B2] Armaleo D. Sun X. Culberson C. (2011). Insights from the first putative biosynthetic gene cluster for a lichen depside and depsidone. Mycologia 103, 741–754. 10.3852/10-335 21289108

[B3] Bergmann S. Schumann J. Scherlach K. Lange C. Brakhage A. A. Hertweck C. (2007). Genomics-driven discovery of PKS-NRPS hybrid metabolites from *Aspergillus nidulans*. Nat. Chem. Biol. 3, 213–217. 10.1038/nchembio869 17369821

[B4] Blatzer M. Gsaller F. Abt B. Schrettl M. Specht T. Haas H. (2014). An endogenous promoter for conditional gene expression in *Acremonium chrysogenum*: the xylan and xylose inducible promoter *xyl1*(P.). J. Biotechnol. 169, 82–86. 10.1016/j.jbiotec.2013.11.003 24246269

[B5] Blumhoff M. Steiger M. G. Marx H. Mattanovich D. Sauer M. (2013). Six novel constitutive promoters for metabolic engineering of *Aspergillus niger*. Appl. Microbiol. Biotechnol. 97, 259–267. 10.1007/s00253-012-4207-9 22707054

[B6] Bok J. W. Chung D. Balajee S. A. Marr K. A. Andes D. Nielsen K. F. (2006). GliZ, a transcriptional regulator of gliotoxin biosynthesis, contributes to *Aspergillus fumigatus* virulence. Infect. Immun. 74, 6761–6768 10.1128/IAI.00780-06 17030582 PMC1698057

[B7] Brakhage A. A. (1997). Molecular regulation of penicillin biosynthesis in *Aspergillus* (*Emericella*) *nidulans*. FEMS Microbiol. Lett. 148, 1–10. 10.1111/j.1574-6968.1997.tb10258.x 9066103

[B8] Cahuzac B. Cerdan R. Felenbok B. Guittet E. (2001). The solution structure of an AlcR-DNA complex sheds light onto the unique tight and monomeric DNA binding of a Zn(2)Cys(6) protein. Structure 9, 827–836. 10.1016/S0969-2126(01)00640-2 11566132

[B9] Calam C. T. Oxford A. E. Raistrick H. (1939). Studies in the biochemistry of micro-organisms: itaconic acid, a metabolic product of a strain of *Aspergillus terreus* Thom. Biochem. J. 33, 1488–1495. 16747058 10.1042/bj0331488 PMC1264600

[B10] Chakravarty A. Carlson J. M. Khetani R. S. Gross R. H. (2007). A novel ensemble learning method for *de novo* computational identification of DNA binding sites. BMC Bioinform. 8:249 10.1186/1471-2105-8-249 PMC1950314 17626633

[B11] Du L. Lou L. (2010). PKS and NRPS release mechanisms. Nat. Prod. Rep. 27, 255–278. 10.1039/b912037h 20111804

[B12] Felenbok B. Sequeval D. Mathieu M. Sibley S. Gwynne D. I. Davies R. W. (1988). The ethanol regulon in *Aspergillus nidulans*: characterization and sequence of the positive regulatory gene *alcR*. Gene 73, 385–396. 10.1016/0378-1119(88)90503-3 3072264

[B13] Fitzgerald M. X. Rojas J. R. Kim J. M. Kohlhaw G. B. Marmorstein R. (2006). Structure of a Leu3-DNA complex: recognition of everted CGG half-sites by a Zn2Cys6 binuclear cluster protein. Structure 14, 725–735. 10.1016/j.str.2005.11.025 16615914

[B14] Fleissner A. Dersch P. (2010). Expression and export: recombinant protein production systems for *Aspergillus*. Appl. Microbiol. Biotechnol. 87, 1255–1270. 10.1007/s00253-010-2672-6 20532762

[B15] Forseth R. R. Fox E. M. Chung D. Howlett B. J. Keller N. P. Schroeder F. C. (2011). Identification of cryptic products of the gliotoxin gene cluster using NMR-based comparative metabolomics and a model for gliotoxin biosynthesis. J. Am. Chem. Soc. 133, 9678–9681. 10.1021/ja2029987 21612254 PMC3151163

[B16] Gressler M. Zaehle C. Scherlach K. Hertweck C. Brock M. (2011). Multifactorial induction of an orphan PKS-NRPS gene cluster in *Aspergillus terreus*. Chem. Biol. 18, 198–209. 10.1016/j.chembiol.2010.12.011 21236704

[B17] Heger A. Holm L. (2000). Rapid automatic detection and alignment of repeats in protein sequences. Proteins 41, 224–237. 10.1002/1097-0134(20001101)41:2<224::AID-PROT70>3.0.CO;2-Z 10966575

[B18] Hutchinson C. R. Kennedy J. Park C. Kendrew S. Auclair K. Vederas J. (2000). Aspects of the biosynthesis of non-aromatic fungal polyketides by iterative polyketide synthases. Antonie Van Leeuwenhoek 78, 287–295. 10.1023/A:1010294330190 11386351

[B19] Klement T. Buchs J. (2013). Itaconic acid–a biotechnological process in change. Bioresour. Technol. 135, 422–431. 10.1016/j.biortech.2012.11.141 23298766

[B20] Lackner G. Bohnert M. Wick J. Hoffmeister D. (2013). Assembly of melleolide antibiotics involves a polyketide synthase with cross-coupling activity. Chem. Biol. 20, 1101–1106. 10.1016/j.chembiol.2013.07.009 23993460

[B21] Lenouvel F. Nikolaev I. Felenbok B. (1997). *In vitro* recognition of specific DNA targets by AlcR, a zinc binuclear cluster activator different from the other proteins of this class. J. Biol. Chem. 272, 15521–15526. 10.1074/jbc.272.24.15521 9182587

[B22] Liu B. H. Chu F. S. (1998). Regulation of *aflR* and its product, AflR, associated with aflatoxin biosynthesis. Appl. Environ. Microbiol. 64, 3718–3723. 9758790 10.1128/aem.64.10.3718-3723.1998 PMC106529

[B23] Liu L. Liu J. Qiu R. X. Zhu X. G. Dong Z. Y. Tang G. M. (2003). Improving heterologous gene expression in *Aspergillus niger* by introducing multiple copies of protein-binding sequence containing CCAAT to the promoter. Lett. Appl. Microbiol. 36, 358–361. 10.1046/j.1472-765X.2003.01321.x 12753242

[B24] Lubertozzi D. Keasling J. D. (2009). Developing *Aspergillus* as a host for heterologous expression. Biotechnol. Adv. 27, 53–75. 10.1016/j.biotechadv.2008.09.001 18840517

[B25] MacPherson S. Larochelle M. Turcotte B. (2006). A fungal family of transcriptional regulators: the zinc cluster proteins. Microbiol. Mol. Biol. Rev. 70, 583–604. 10.1128/MMBR.00015-06 16959962 PMC1594591

[B26] Menne S. Walz M. Kuck U. (1994). Expression studies with the bidirectional *pcbAB*-*pcbC* promoter region from *Acremonium chrysogenum* using reporter gene fusions. Appl. Microbiol. Biotechnol. 42, 57–66. 10.1007/BF00170225 7765820

[B27] Meyer V. Wanka F. Van Gent J. Arentshorst M. Van Den Hondel C. A. Ram A. F. (2011). Fungal gene expression on demand: an inducible, tunable, and metabolism-independent expression system for *Aspergillus niger*. Appl. Environ. Microbiol. 77, 2975–2983. 10.1128/AEM.02740-10 21378046 PMC3126388

[B28] Minetoki T. Kumagai C. Gomi K. Kitamoto K. Takahashi K. (1998). Improvement of promoter activity by the introduction of multiple copies of the conserved region III sequence, involved in the efficient expression of *Aspergillus oryzae* amylase-encoding genes. Appl. Microbiol. Biotechnol. 50, 459–467. 10.1007/s002530051321 9830097

[B29] Nikolaev I. Cochet M. F. Lenouvel F. Felenbok B. (1999). A single amino acid, outside the AlcR zinc binuclear cluster, is involved in DNA binding and in transcriptional regulation of the *alc* genes in *Aspergillus nidulans*. Mol. Microbiol. 31, 1115–1124. 10.1046/j.1365-2958.1999.01250.x 10096079

[B30] Pachlinger R. Mitterbauer R. Adam G. Strauss J. (2005). Metabolically independent and accurately adjustable *Aspergillus* sp. expression system. Appl. Environ. Microbiol. 71, 672–678. 10.1128/AEM.71.2.672-678.2005 15691916 PMC546773

[B31] Parrot D. Peresse T. Hitti E. Carrie D. Grube M. Tomasi S. (2015). Qualitative and spatial metabolite profiling of lichens by a LC-MS approach combined with optimised extraction. Phytochem. Anal. 26, 23–33. 10.1002/pca.2532 25130294

[B32] Punt P. J. Dingemanse M. A. Kuyvenhoven A. Soede R. D. Pouwels P. H. Van Den Hondel C. A. (1990). Functional elements in the promoter region of the *Aspergillus nidulans gpdA* gene encoding glyceraldehyde-3-phosphate dehydrogenase. Gene 93, 101–109. 10.1016/0378-1119(90)90142-E 2121607

[B33] Schroeckh V. Scherlach K. Nutzmann H. W. Shelest E. Schmidt-Heck W. Schuemann J. (2009). Intimate bacterial-fungal interaction triggers biosynthesis of archetypal polyketides in *Aspergillus nidulans*. Proc. Natl. Acad. Sci. U.S.A. 106, 14558–14563 10.1073/pnas.0901870106 19666480 PMC2732885

[B34] Sharma R. Katoch M. Srivastava P. S. Qazi G. N. (2009). Approaches for refining heterologous protein production in ?lamentous fungi. World J. Microbiol. Biotechnol. 25, 2083–2094 10.1007/s11274-009-0128-x

[B35] Shoji J. Y. Maruyama J. Arioka M. Kitamoto K. (2005). Development of *Aspergillus oryzae thiA* promoter as a tool for molecular biological studies. FEMS Microbiol. Lett. 244, 41–46. 10.1016/j.femsle.2005.01.014 15727819

[B36] Slesiona S. Gressler M. Mihlan M. Zaehle C. Schaller M. Barz D. (2012a). Persistence *versus* escape: *Aspergillus terreus* and *Aspergillus fumigatus* employ different strategies during interactions with macrophages. PLoS ONE 7:e31223 10.1371/journal.pone.0031223 22319619 PMC3272006

[B37] Slesiona S. Ibrahim-Granet O. Olias P. Brock M. Jacobsen I. D. (2012b). Murine infection models for *Aspergillus terreus* pulmonary aspergillosis reveal long-term persistence of conidia and liver degeneration. J. Infect. Dis. 205, 1268–1277. 10.1093/infdis/jis193 22438397

[B38] Todd R. B. Andrianopoulos A. Davis M. A. Hynes M. J. (1998). FacB, the *Aspergillus nidulans* activator of acetate utilization genes, binds dissimilar DNA sequences. Embo J. 17, 2042–2054. 10.1093/emboj/17.7.2042 9524126 PMC1170549

[B39] Verdoes J. C. Punt P. J. Schrickx J. M. Van Verseveld H. W. Stouthamer A. H. Van Den Hondel C. A. (1993). Glucoamylase overexpression in *Aspergillus niger*: molecular genetic analysis of strains containing multiple copies of the *glaA* gene. Transgenic Res. 2, 84–92. 10.1007/BF01969381 8513339

[B40] Vogt K. Bhabhra R. Rhodes J. C. Askew D. S. (2005). Doxycycline-regulated gene expression in the opportunistic fungal pathogen *Aspergillus fumigatus*. BMC Microbiol. 5:1. 10.1186/1471-2180-5-1 15649330 PMC546209

[B41] Ward O. P. (2012). Production of recombinant proteins by filamentous fungi. Biotechnol. Adv. 30, 1119–1139. 10.1016/j.biotechadv.2011.09.012 21968147

[B42] Xu B. Yin Y. Zhang F. Li Z. Wang L. (2012). Operating conditions optimization for (+)-terrein production in a stirred bioreactor by *Aspergillus terreus* strain PF-26 from marine sponge *Phakellia fusca*. Bioprocess Biosyst. Eng. 35, 1651–1655. 10.1007/s00449-012-0735-z 22527032

[B43] Xu Y. Espinosa-Artiles P. Schubert V. Xu Y. M. Zhang W. Lin M.. (2013). Characterization of the biosynthetic genes for 10,11-dehydrocurvularin, a heat shock response-modulating anticancer fungal polyketide from *Aspergillus terreus*. Appl. Environ. Microbiol. 79, 2038–2047. 10.1128/AEM.03334-12 23335766 PMC3592213

[B44] Yin Y. Xu B. Li Z. Zhang B. (2012). Enhanced production of (+)-terrein in fed-batch cultivation of *Aspergillus terreus* strain PF26 with sodium citrate. World J. Microbiol. Biotechnol. 29, 441–446. 10.1007/s11274-012-1196-x 23085955

[B45] Yu J. Chang P. K. Ehrlich K. C. Cary J. W. Bhatnagar D. Cleveland T. E.. (2004). Clustered pathway genes in aflatoxin biosynthesis. Appl. Environ. Microbiol. 70, 1253–1262. 10.1128/AEM.70.3.1253-1262.2004 15006741 PMC368384

[B46] Yu J. H. Butchko R. A. Fernandes M. Keller N. P. Leonard T. J. Adams T. H. (1996). Conservation of structure and function of the aflatoxin regulatory gene *aflR* from *Aspergillus nidulans* and *A. flavus*. Curr. Genet. 29, 549–555. 10.1007/BF02426959 8662194

[B47] Zaehle C. Gressler M. Shelest E. Geib E. Hertweck C. Brock M. (2014). Terrein biosynthesis in *Aspergillus terreus* and its impact on phytotoxicity. Chem. Biol. 21, 719–731. 10.1016/j.chembiol.2014.03.010 24816227

