# Supplementary material for: A new high-performance heterologous fungal expression system based on regulatory elements from the Aspergillus terreus terrein gene cluster
Source: Front Microbiol. 2015 Mar 16;6:184. doi: 10.3389/fmicb.2015.00184 (PMC4360782; doi:10.3389/fmicb.2015.00184)
Supplement: Supplementary file 1 [file DataSheet1.DOC]

***Supplementary Material***

**A new high-performance heterologous fungal expression system based on regulatory elements from the *Aspergillus terreus* terrein gene cluster**

**Markus Gressler^1^, Peter Hortschansky^2^, Elena Geib^1^ and Matthias Brock^1,3^***

^1^ Microbial Biochemistry and Physiology, Leibniz Institute for Natural Product Research and Infection Biology, -Hans Knoell Institute-, Beutenbergstr. 11a, 07745 Jena, Germany

^2^ Molecular and Applied Microbiology, Leibniz Institute for Natural Product Research and Infection Biology, -Hans Knoell Institute-, Beutenbergstr. 11a, 07745 Jena, Germany

^3^ Institute for Microbiology, Friedrich Schiller University, 07743 Jena, Germany

*** Correspondence:** Matthias Brock, Microbial Biochemistry and Physiology, Leibniz Institute for Natural Product Research and Infection Biology, -Hans Knoell Institute-, Beutenbergstr. 11a, 07745 Jena, Germany,

Matthias.brock@hki-jena.de

1. **Supplementary Experimental Procedures**

**1.1 Generation of the β-galactosidase reporter strain *A. niger* A1144_P*terA*:*lacZ***

For all PCR reactions the high-fidelity proofreading Phusion polymerase was used (Thermo Biosciences). To generate an *A. niger* β-galactosidase reporter strain that expresses the *E. coli lacZ* gene under control of the *A. terreus terA* promoter (P*terA*; 1220 bp), the promoter fragment was amplified from genomic DNA of *A. terreus* SBUG844 with oligonucleotides P36/37, cloned in the pJET1.2 vector (Thermo Biosciences) and subcloned into the *NotI*/*BamHI* digested plasmid *lacZ*:*trpC*T-pJET1.2 . The plasmid was linearized by *NotI* digestion and the *ptrA*-cassette from plasmid ptrA-pJET1 was inserted. The plasmid was used for transformation of *A. niger* A1144 resulting in A1144_P*terA*:*lacZ*. Transformants with two genomic integrations of the construct were selected for downstream investigations.

**1.2 Expression of *terR* in *A. niger* A1144, *A. niger* A1144_P*terA*:*lacZ* and overexpression in *A. terreus* SBUG844**

For expression of *terR* under control of its native promoter, the region spanning the promoter, coding sequence and its terminator (*terR*+P+T; 4130 bp) was amplified with oligonucleotides P38/39 from genomic DNA of *A. terreus* SBUG844 and cloned into pJET1.2. The plasmid was linearized by *NotI* digestion and the *hph* resistance cassette from *hph*-pCRIV was inserted. The resulting vector hph_P*terR*:*terR*_pJET1.2 was used for transformation of A1144_P*terA*:*lacZ.*

For overexpression of *terR*, the coding region of *terR* (ATEG_00139) together with its terminator (3032 bp) was amplified from genomic DNA of *A. terreus* SBUG844 with oligonucleotides P40/39 and cloned into the pJET1.2 vector resulting in plasmid 00139+T-pJET1.2. The promoter of the glyceraldehyde-3-phosphate dehydrogenase from *A. nidulans* (AnP*gpdA*; 1336 bp) and the α-amylase B promoter from *A. oryzae* (AoP*amyB*; 1019 bp) were amplified with oligonucleotides P41/42 from genomic DNA of *A. nidulans* FGSC A4 gDNA and with oligonucleotides P43/44 from genomic DNA of *A. oryzae* FGSC A815, respectively. Fragments were subcloned into pJET1.2, excised by *BamHI/NotI* restriction and subcloned into plasmid 00139+T-pJET1.2. The *hph* resistance cassette from *hph*-pCRIV was inserted into the *NotI*-linerarized vectors. The resulting plasmids *hph*_AnP*gpdA*:*terR*_pJET1.2 and *hph*_AoP*amyB*:*terR*_pJET1.2 were used for transformation of *A. niger* A1144_P*terA*:*lacZ*, *A. niger* A1144_P*terA*:*lacZ* or *A. terreus* SBUG844 as specified in the respective experiments. For transformation of *A. niger* A1144, the *hph* cassette of plasmid *hph*_AoP*amyB*:*terR*_pJET1.2was replaced by the *ptrA* resistance cassette. Transformants were analyzed by Southern blot and strains that contained single or double integrations of the constructs were selected as specified.

**1.3 Construction of P*terC*:*lacZ* and P*terC*m_1/2_:*lacZ* reporter strains in *A. terreus* SBUG844**

All sequences of the *terC* promoter were amplified from genomic DNA of *A. terreus* SBUG844. To generate the P*terC*:*lacZ* reporter, the promoter (P*terC*; 1203 bp) was amplified with oligonucleotides P45/46. To exchange the putative “BS4” by “BS5” (P*terC*m1) the upstream and downstream promoter fragments adjacent to “BS4” were amplified with oligonucleotides P45/47 and P48/46, gel purified and fused by overlap-PCR using the flanking oligonucleotides P45/46 generating the P*terC*m1 fragment. After subcloning into pJET1.2, the native and mutated P*terC* fragments were excised by *Not*I/*BamH*I restriction and ligated into the *Not*I/*BamH*I digested plasmid *lacZ*:*trpC*T-pJET1.2 . The plasmids were linearized with *Not*I and the *ptrA* resistance cassette was inserted. To delete the two CGG half-sites of “BS4” in P*terC* (P*terC*m2), a 928 bp fragment of the *terC* promoter was excised by *Stu*I/*Sma*I restriction from plasmid *ptrA_*P*terC*:*lacZ*:*trpC*^T^-pJET1.2. Two fragments containing the mutated half-sites were amplified with oligonucleotides P49/50 and P51/52 and inserted into the *Stu*I/*Sma*I restricted vector by *in vitro* recombination using the InFusion Kit HD cloning kit (Clonetech laboratories). All three reporter plasmids were used for transformation of *A. terreus* SBUG844 resulting in strains SBUG844_P*terC*:*lacZ*, SBUG844_P*terC*m1:*lacZ* and SBUG844_P*terC*m2:*lacZ*. Independent transformants with single copy integration of the respective construct were randomly selected for further investigations.

**1.4 Generation of reporter strains to investigate the bi-directionality of the *terA*/*B* promoter**

The *tdTomato* gene (1445 bp) was amplified from *tdTom*-pUC57 with oligonucleotides P53/54 and cloned into pJET1.2. The *tdTomato* gene was excised by *Not*I/*BamH*I restriction and ligated into the *Not*I/*BamH*I restricted plasmid *lacZ*:*trpC*^T^_pJET1.2. The resulting plasmid *tdTom*:*lacZ*:*trpC*^T^_pJET1.2 was linearized with *Not*I and the *hph* resistance cassette was inserted. The *BamH*I restriction site between the *lacZ* and *tdTomato* gene enabled the subsequent insertion of the bi-directional *terA/B* promoter in both orientations. For this purpose, the 783 bp intergenic region between *terA* and *terB* was amplified with oligonucleotides P55/56 from genomic DNA of *A. terreus* SBUG844 resulting in terminal *Bgl*II restriction sites at the 5’- and 3’-flank. The fragment was cloned into pJET1.2, excised with *Bgl*II ligated into the *BamH*I restricted plasmid *hph*_*tdTomato*:*lacZ*:*trpC*^T^_pJET1.2. Orientation of the promoter was checked by PCR and plasmids containing the promoter in both orientations were used for transformation of A1144_P*amyB*:*terR* (P2). Depending on the orientation of the promoter this resulted in strains *A. niger* A1144_AoP*amyB*:*terR*_P*terA*:*lacZ*_P*terB*:*tdTom* and *A. niger* A1144_AoP*amyB*:*terR*_P*terB*:*lacZ*_P*terA*:*tdTom*. Independent transformants with single copy integration of the respective construct were selected for further investigations.

**1.5 Generation of reference reporter strains *A. niger* A1144_AnP*gpdA*:*lacZ* and A1144_AnP*gpdA*:*tdTom***

To generate the β-galactosidase reference reporter strains, the *gpdA* promotor from *A. nidulans* was excised from AnP*gpdA*-JET1.2 by *Bgl*II/*Hind*III restriction and was cloned into the *BamH*I/*Hind*III restricted vector *lacZ*:*trpC*^T^-pJET1.2. After restriction with *Not*I the *ptrA* resistance cassette was introduced. To generate the fluorescent tdTomato reference reporter strain, the *tdTomato* gene (1437 bp) was with excised *Bgl*II/*Hind*III from *tdTom*-pUC57 and cloned into AnP*gpdA*-JET1.2. The plasmid AnP*gpdA*:*tdTom*-pJET1.2 was linearized with *Not*I and the *hph* resistance cassette was inserted. Both plasmids were used for transformation of the *A. niger* A1144 wild type. Independent transformants with single copy integration of the respective constructs were selected as reference strains.

**1.6 Heterologous expression of polyketide synthase genes in P2**

For heterologous expression of the *terA* gene (ATEG_00145) the entire coding region including its promoter and terminator sequence were amplified with oligonucleotides P57/56 (7676 bp) from genomic DNA of *A. terreus* SBUG844. The fragment was ligated into pJET1.2/blunt. The plasmid was linearized with *Not*I and the *hph* resistance cassette was inserted. The resulting plasmid P*terA*:*terA:terA*^T^_*hph*_pJET1.2 was used for transformation of A1144_P*amyB*:*terR* (P2). For heterologous expression of the *orsA* gene (AN7909) under control of the *terA* promoter the coding region of *orsA* together with its terminator was amplified from genomic DNA of *A. nidulans* FGSC A4 using oligonucleotides P58/59 (6707 bp). The *terA* promoter was amplified from genomic DNA of *A. terreus* SBUG844 with oligonucleotides P60/61 (776 bp). The promoter and the *orsA* fragments were fused and introduced into the *EcoR*V digested *hph*-pCRIV vector by *in vitro* recombination using the InFusion HD Cloning Kit (Clonetech Laboratories). The resulting plasmid P*terA*:*orsA*:*orsA*^T^_*hph*_pCRIV was used for transformation of A1144_P*amyB*:*terR* (P2). For heterologous expression of the *orsA* gene with a domain swapping of the thioesterase domain from *terA,* two different constructs were generated, since the starting position of thioesterase domains has not been well defined yet. The *orsA* gene (AN7909) without its sequence coding for the TE domain was amplified from genomic DNA of *A. nidulans* FGSC A4 using either oligonucleotides P62/63 (5873 bp) or P62/64 (5389 bp). In accordance, the sequence coding for the TE domain from *terA* (ATEG_00145) was amplified from genomic DNA of *A. terreus* SBUG844 with either oligonucleotides P65/66 (597 bp) or P67/66 (1126 bp). The expression vector P*terA*:*trpC*^T^_AnP*gpdA*:*ble*:*trpC*^T^_pUC19 (SM Xpress) was linearized with *Nco*I and the fragments were ligated by *in vitro* recombination using the InFusion HD Cloning Kit (Clontech). The resulting plasmids contained the *orsA* gene with TE domain from *terA* under transcriptional control of the *terA* promoter and were used for transformation of *A. niger* A1144_P*amyB*:*terR* (P2). Transformants with single copy integrations of the constructs were selected for metabolite identification.

**2. Supplementary Figures and Tables**

## 2.1 Supplementary Tables

# Table S1. Wild type strains and mutants from this study

| **Strain** | **Genotype** | **Reference** |
| --- | --- | --- |
| FGSC A1144 | wild type | FGSC; Kansas City; USA |
| FGSC A4 | wild type | FGSC; Kansas City; USA |
| SBUG844 | wild type | JMRC, HKI; Jena; Germany |
| SBUG844Δ*akuB* | Δ*akuB*::*hph* | Gressler *et al.*, 2011 |
| SBUG844Δ*akuB*Δ*terR* | Δ*akuB*::*hph*; Δ*terR*::*ptrA* | Zaehle *et al*., 2014 |
| SBUG844_AnP*gpdA*:*terR* | *hph*, AnP*gpdA*:*terR:terR*^T^ | This study. |
| SBUG844_P*terC*:*lacZ* | *ptrA*, P*terC*:*lacZ*:*trpC*^T^ | This study. |
| SBUG844_P*terC*m1:*lacZ* | *ptrA*, P*terC*m1:*lacZ*:*trpC*^T^ | This study. |
| SBUG844_P*terC*m2:*lacZ* | *ptrA*, P*terC*m2:*lacZ*:*trpC*^T^ | This study. |
| FGSC A1144_P*terA*:*lacZ* | *ptrA*, P*terA*:*lacZ*:*trpC*^T^ | This study. |
| FGSC A1144_P*terA*:*lacZ_*P*terR*:*terR* | *ptrA*, P*terA*:*lacZ*:*trpC*^T^;  *hph*, P*terR*:*terR*:*terR*^T^ | This study. |
| FGSC A1144_P*terA*:*lacZ_*AnP*gpdA*:*terR* | *ptrA*, P*terA*:*lacZ*:*trpC*^T^;  *hph*, AnP*gpdA*:*terR*:*terR*^T^ | This study. |
| FGSC A1144_P*terA*:*lacZ_*AoP*amyB*:*terR* | *ptrA*, P*terA*:*lacZ*:*trpC*^T^;  *hph*, AoP*amyB*:*terR*:*terR*^T^ | This study. |
| FGSC A1144_AoP*amyB*:*terR* | *ptrA*, AoP*amyB*:*terR*:*terR*^T^ | This study. |
| FGSC A1144 AoP*amyB*:*terR_PterA:lacZ_PterB:tdTom* | *ptrA*, AoP*amyB*:*terR*:*terR*^T^; *hph*, P*terA:lacZ:trpC*^T^, *PterB*:*tdTom* | This study. |
| FGSC A1144 AoP*amyB*:*terR_PterB:lacZ_PterA:tdTom* | *ptrA*, AoP*amyB*:*terR*:*terR*^T^; *hph*, P*terB:lacZ:trpC*^T^, *PterA*:*tdTom* | This study. |
| FGSC A1144_AnP*gpdA*:*lacZ* | *ptrA*, AnP*gpdA:lacZ:trpC*^T^ | This study. |
| FGSC A1144_AnP*gpdA*:*tdTom* | *hph*, AnP*gpdA:tdTom* | This study. |
| FGSC A1144_AoP*amyB*:*terR_PterA:terA* | *ptrA*, AoP*amyB*:*terR*:*terR*^T^;  *hph*, P*terA:terA:terA*^T^ | This study. |
| FGSC A1144_AoP*amyB*:*terR_PterA:orsA* | *ptrA*, AoP*amyB*:*terR*:*terR*^T^;  *hph*, P*terA:orsA:orsA*^T^ | This study. |
| FGSC A1144 AoP*amyB*:*terR_PterA*:*orsA*:TE*terA_partial:trpC*^T^ | *ptrA*, AoP*amyB*:*terR*:*terR*^T^;  *hph*, P*terA:orsA*:TE*terA_partial:trpC*^T^ | This study. |
| FGSC A1144 AoP*amyB*:*terR_PterA*:*orsA*:TE*terA_complete:trpC*^T^ | *ptrA*, AoP*amyB*:*terR*:*terR*^T^; *hph*, P*terA:orsA*:TE*terA_complete:trpC*^T^ | This study. |
| BL21(DE3) Rosetta2 | *fhuA2 [lon] ompT gal (λ DE3) [dcm] ∆hsdS*  *λ DE3 = λ sBamHIo ∆EcoRI-B int::(lacI::PlacUV5::T7 gene1) i21 ∆nin5* with plasmid pRARE2 | Novagen, Germany |
| BL21(DE3)_P*lacZ*:*terR*_1-153_ | BL21(DE3) Rosetta2 genotype with P*lacZ*:*terR*_1-153_-pET29a | This study. |
| BL21(DE3)_P*lacZ*:*terR*_35-138_ | BL21(DE3) Rosetta2 genotype with P*lacZ*:*terR*_35-138_-pET29a | This study. |
| BL21(DE3)_P*lacZ*:*terR*_43-138_ | BL21(DE3) Rosetta2 genotype with P*lacZ*:*terR*_43-138_-pET29a | This study. |

**Table S2. Oligonucleotides used in this study.**

| **No.** | **name** | **5´-3´sequence** |
| --- | --- | --- |
| P1 | terR_NdeI_f | catatgttcgccgaacttaacgcaaag |
| P2 | terR_Hind_r | aagcttagctatgccggtcatttgtg |
| P3 | Nde_shortTerR_f | catatgtctcggcgcagaggtg |
| P4 | Bam_shortTerR_r | ggatcctcagcccgcatttccggaacc |
| P5 | TerR35-138_f | catatgtcttctgtccggaaaaagaagtg |
| P6 | EcoRI_PterA_for | gacggccagtgaattcgatcctctctctgatattgtcg |
| P7 | NcoI_PterA_rev | ccatggtgctgtgatgagaagtttg |
| P8 | NcoI_TrpCT_for | atcacagcaccatggcagcagtgatttcaatctgaacc |
| P9 | EcoRI_TrpCT_rev | taccgagctcgaattcgagtgagggttgagtacgag |
| P10 | At00135_for1 | gaatggatatcgctggatgc |
| P11 | At00135_rev1 | ccaaggcatcactattatcgac |
| P12 | At00136_rev1 | gtccaaacgatccaaggtgg |
| P13 | At00136_for1 | ggtcttacagcttctcctacc |
| P14 | At00137_for1 | cgtctattctgcactcaaagc |
| P15 | At00137_rev1 | ctgtcctcaaagctcgtcc |
| P16 | At00138_for1 | gtggctttctcctatcctcg |
| P17 | At00138_rev1 | ctcttccacttgagtcctgg |
| P18 | At00139_for1 | caagaagaagcatgtggtacc |
| P19 | At00139_rev1 | gatggccaacacagcttgg |
| P20 | At00140_for1 | cctacacaaacatcacctacg |
| P21 | At00140_rev1 | ggaacgtctgaacgactgg |
| P22 | At00141_for1 | gattacgagaccacggtgc |
| P23 | At00141_rev1 | gatagccttccaaagactcc |
| P24 | At00142_for1 | cagacgtgctgattaactgg |
| P25 | At00142_rev1 | cacaatgacctcgatcaagc |
| P26 | At00143_for1 | gtacgcaagtcgctgtttgg |
| P27 | At00143_rev1 | cacctcgtcgtacttgtcc |
| P28 | At00144_for2 | cgatgaatgtcagcctgagc |
| P29 | At00144_rev2 | gatcaaaggcaaatatacgtacc |
| P30 | At00145_for2 | ggaatcgaaggtgttgctgc |
| P31 | At00145_rev2 | cctatcaacttctcccatcc |
| P32 | act_Ater_for | ccatcgagaagtcttatgagc |
| P33 | act_Ater_rev | ggacagggaagccagaatgg |
| P34 | Anig_gpdA_for | caagttcggcatcgttgagg |
| P35 | Anig_gpdA_rev | ccactcgttgtcgtaccagg |
| P36 | NotI_P00145_f | gcggccgcaatatttgtgtgtcgagaacc |
| P37 | BglII_P00145_r | agatctcatggtgctgtgatgagaagtttg |
| P38 | NotI_00140in_for | ttggtgcggccgcatctatcgggacatgttg |
| P39 | SwaI00139OE_rev | atttaaatcatggtagtcaggttgtgc |
| P40 | BamHI_00139OE_f | ggatccttcgccgaacttaacgcaaagg |
| P41 | NotI_AnPgpdA_for | gcggccgctcaccacaaaagtcagacg |
| P42 | BamHI_AnPgpdA_rev | ggatcccattgtgatgtctgctcaagc |
| P43 | NotI_AoPamyB_for | gcggccgctacttaaaaatcgatctcgcag |
| P44 | BamHI_AoPamyB_rev | ggatcccataaatgccttctgtggggtt |
| P45 | NotI_P00143_for | gcggccgcatgtggaagattagtgg |
| P46 | BamHI_P00143_rev | ggatcccatttgtatgccaggagcaaag |
| P47 | SwitchBS4-5_rev | ggcggctcctcggtacagcgaatgcacgccaccccggctagc |
| P48 | SwitchBS4-5new_f | ccgaggagccgcccccaggaatggaaatccggcctccg |
| P49 | StuI_PterCmut3_f | cgtcatacaccaaggccttgtcttatcttccatatgagg |
| P50 | BSdel_PterCmut3_r | caacaatacaaactcgggaatgcacgccacc |
| P51 | BSdel_PterCmut3_f | gagtttgtattgttgaagaggtcaatggaaatccggcctc |
| P52 | SmaI_PterCmut3_r | catgtatgccagcccgggccaggactaggctcaagg |
| P53 | BamHI_tdTom_for | ggatccatggtctccaagggtgagg |
| P54 | NotI_tdTom_rev | gcggccgcctacttgtagagctcgtccatac |
| P55 | BglII_P00145_rev | agatctcatggtgctgtgatgagaagtttg |
| P56 | BglII_P00144_rev | agatctcatgatcctctctctgatattg |
| P57 | SwaI_00145OEdn_rev | atttaaatgagccgtgaactgtatgac |
| P58 | SATorsA_for | atggctccaaatcacgttctttttttcc |
| P59 | pCRIV-TorsA_r | gccagtgtgatggatcgcaaccctgattatccggttaaag |
| P60 | SATorsA_PterA_r | gtgatttggagccatggtgctgtgatgagaagtttg |
| P61 | pCRIV-PterA_f | gcgaattctgcagatgctgtgtctggtatgtgc |
| P62 | PterA_orsA_for | catcacagcaccatggctccaaatcacgttctt |
| P63 | orsA_forTE-S_r | gcccgcggaccaaccgcc |
| P64 | orsA_forTE-L_r | cgagtccatacccaccgag |
| P65 | orsA_TE(terA)S_f | ggttggtccgcgggcggtattttggcgtatgcggtg |
| P66 | trpCT_TEterA_rev | atcactgctgccatggtcatgcaccgatcaagcgatc |
| P67 | orsA_TE(terA)L_f | gtgggtatggactcgttgatgtcacttaccatcactgg |
| P68 | LacZ_up_down | ggcgttacccaacttaatcgc |
| P69 | LacZ_mitte_up | ctcatccatgacctgaccatg |
| P70 | tdTom_in_for1 | ggtcacgagttcgagatcgagg |
| P71 | tdTom_in_rev1 | ggtgtagtcctcgttgtgg |
| P72 | SATorsA_for | atggctccaaatcacgttctttttttcc |
| P73 | terA_SATorsA_r | gttggggaagcggccggacatgccaacgacggcaatg |
| P74 | terA+STOP1_r | caacacctcaaatggccaaccttgcctg |
| P75 | EcoRV_00145_for | gatatcggcatggatgtgcgtg |

**2.2 Supplementary Figures**

**
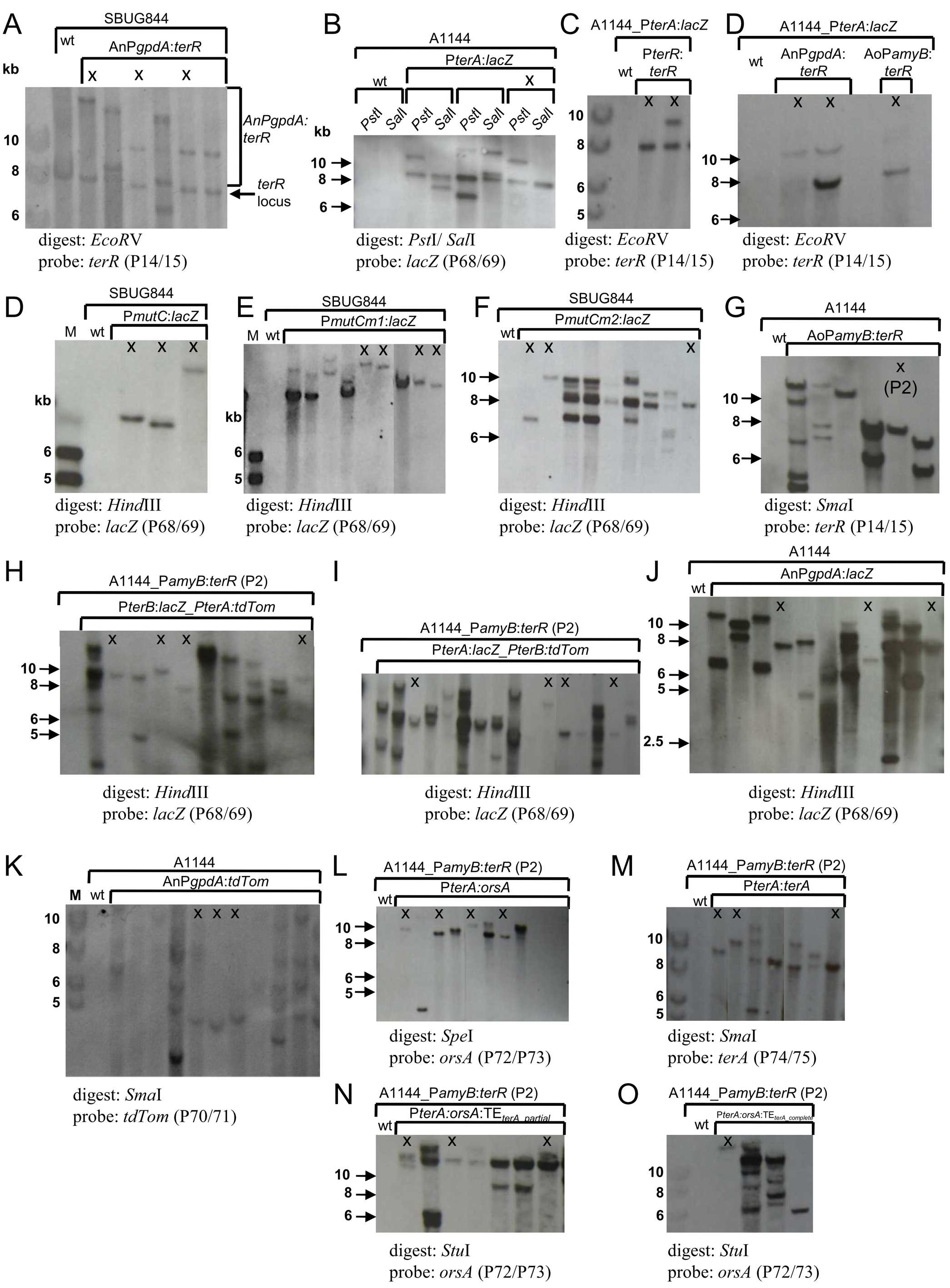
**

**Figure S1.** **Southern Blot analyses for *A. terreus* and *A. niger* mutants generated in this study.** Genomic DNA of parental strains and transformants was restricted with indicated enzymes, blotted on nylon membranes and hybridized with the indicated digoxygenin-labeled probes. Primer pairs used for generation of probes are indicted in brackets and sequences are found in Table S2. Signals were detected by using anti-digoxygenin FAB-fragments conjugated with alkaline phosphatase and the CDP star chemiluminescent substrate (Roche). Strains marked by “X” have been investigated in more detail as described in the main manuscript text. **(A)** Overexpression of *terR* in SBUG844 under control of the *A. nidulans gpdA* promoter. In selected transformants the original *terR* locus (signal at 8125 bp) is not affected. **(B)** Southern blot of the β-galactosidase reporter strain A1144_P*terA*:*lacZ* in the *A. niger* FGSC A1144 background. The selected transformant contains two integrations as revealed from the *PstI* digest. **(C)** Integration of *terR* under its native promoter (P*terR*) in the genome of β-galactosidase reporter strain A1144_P*terA*:*lacZ*. A strain with single and with double integration was selected. **(D-F)** Southern blot of the β-galactosidase reporter strains A1144_P*terC*:*lacZ*, A1144_P*terCm1*:*lacZ,* and A1144_P*terCm2*:*lacZ.* Single copy integrants were selected for determination of reporter activity. **(G)** Southern blot of *A. niger* strains expressing *terR* under control of the *A. oryzae amyB* promoter. The strain P2 was selected for heterologous metabolite production and analysis of transcriptional activity from the bi-directional *terA*/*B* promoter. **(H-I)** Southern blot of transformants expressing the *lacZ* and *tdTomato* gene under control of the bi-directional *terA*/*B* promoter. Constructs are done in both directions. The P2 strain serves as genetic background. **(J)** Southern blot of *A. niger* reference strains (A1144_AnP*gpdA*:*lacZ*) expressing the *lacZ* under control of the *A. nidulans gpdA* promoter. **(K)** Southern blot of *A. niger* reference strains (A1144_AnP*gpdA*:*tdTom*) expressing the *tdTomato* gene under control of the *A. nidulans gpdA* promoter. **(L)** Southern blot of the P2 strain with integration of the P*terA*:*orsA* fusion (P2_P*terA*:*orsA*). **(M)** Southern blot of the P2 strain with integration of the *terA* gene under control of its native *terA* promoter (P2_P*terA*:*terA*) **(N-O)** Southern blot of chimera fusions of *orsA* with partial (N; P2_P*terA*:*orsA*:TEterA_partial) and full-length replacement (P2_P*terA*:*orsA*:TEterA_complete) of the *orsA* thioesterase domain for expression in *A. niger* P2.


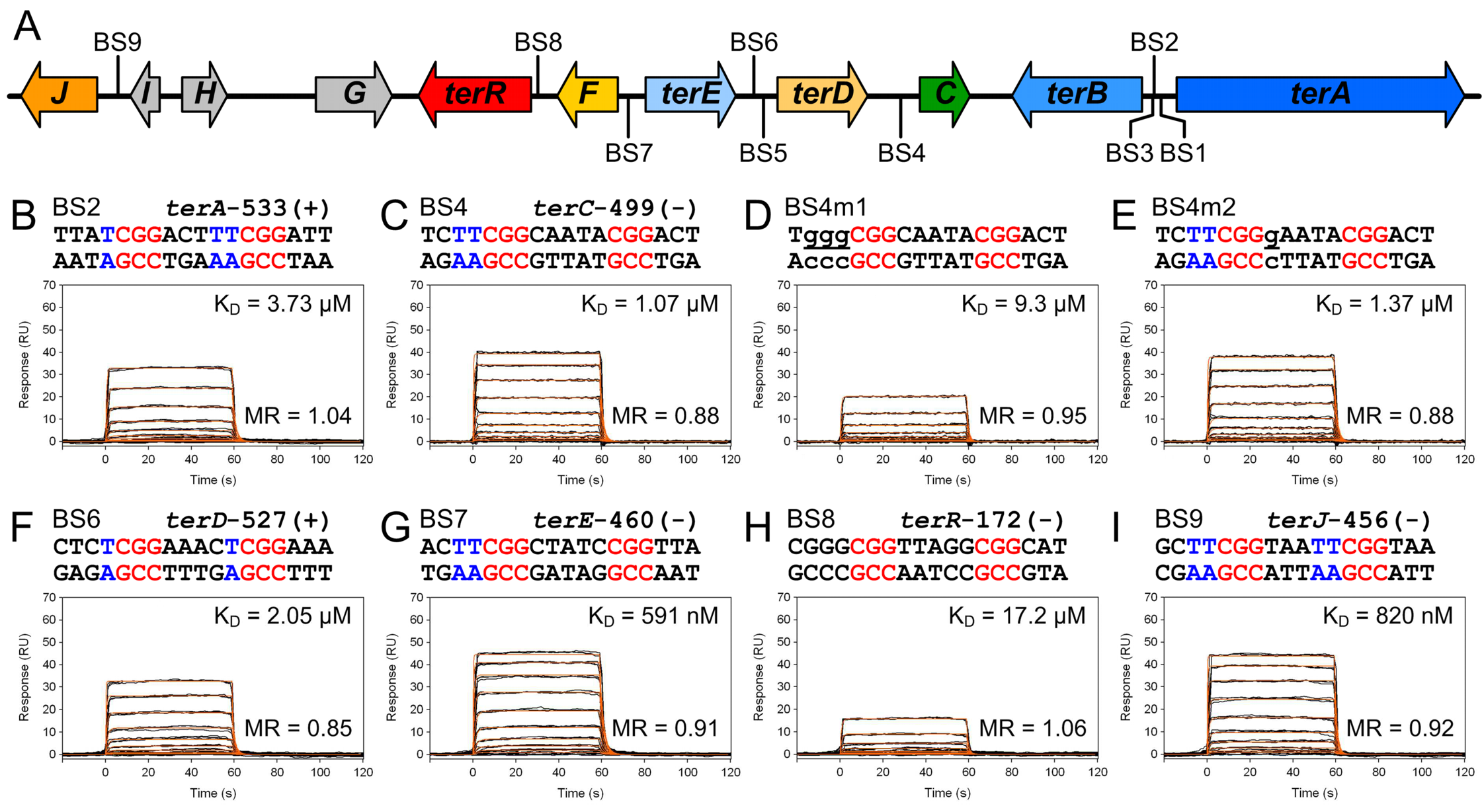


**Figure S2.** **Real-time *in vitro* SPR interaction analysis of TerR_43-138_ with selected predicted native or mutated TerR DNA-binding sites.** The TerR_43-138_ fragment lacks an *N*-terminal basic stretch present in the TerR_35-138_ fragment. Intergenic positions of the consensus TerR-binding motif identified by the SCOPE motif finder suite. Sequences of DNA duplexes used for SPR analysis are shown on top of the sensorgrams. CGG half-sites and 5`-flanking thymidines are highlighted in red and blue. Substituted nucleotides relative to the wild-type sequence are underlined and shown in lowercase letters. TerR_43-138_ binding responses from triplicate injections of different concentrations (black lines) are overlaid with the best fit derived from a 1:1 interaction model including a mass transport term (red lines). Dissociation constants (K_D_) and protein:DNA molar ratios (MR) are plotted inside the sensorgrams. For more details refer to Figure 3 and section “A Basic Stretch at the TerR *N*-terminus Promotes High Affinity DNA Binding” of the main manuscript text.


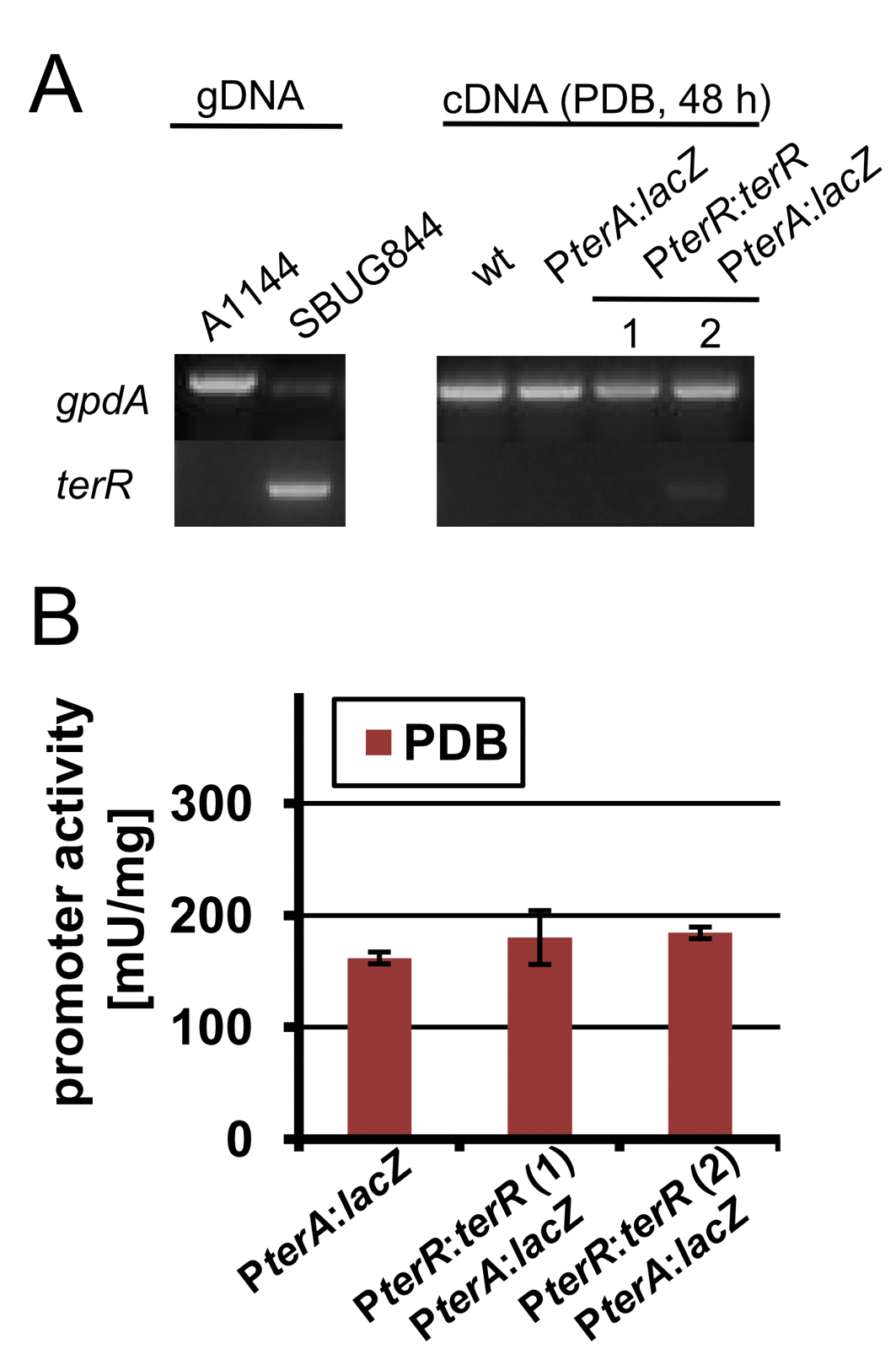


**Figure S3. Semiquantitative RT-PCR analysis and β-galactosidase activity of *A. niger* strains A1144_P*terA*:*lacZ* and A1144_P*terA*:*lacZ*_P*terR*:*terR*.** Strains were grown for 48 h in PDB medium. **(A)** Semi-quantitative PCR analysis. Left panel: genomic DNA from *A. niger* A1144 and *A. terreus* SBUG844 wild-type strains as template for control amplification of the *A. niger gpdA* and *A. terreus terR* gene. Right panel: Analysis of cDNA from *A. niger* wild type strain and strains containing only the P*terA*:*lacZ* reporter construct or the reporter together with *terR* under its native promoter in single (1) or double (2) integration. Under its native promoter, no *terR* transcript is observed. **(B)** Determination of β-galactosidase activity. Strains shown in (A) were analyzed for reporter activity. A low background activity is observed that does not increase in reporter strains that contain the *terR* gene under its native promoter regardless of the number of integrations (1 or 2).


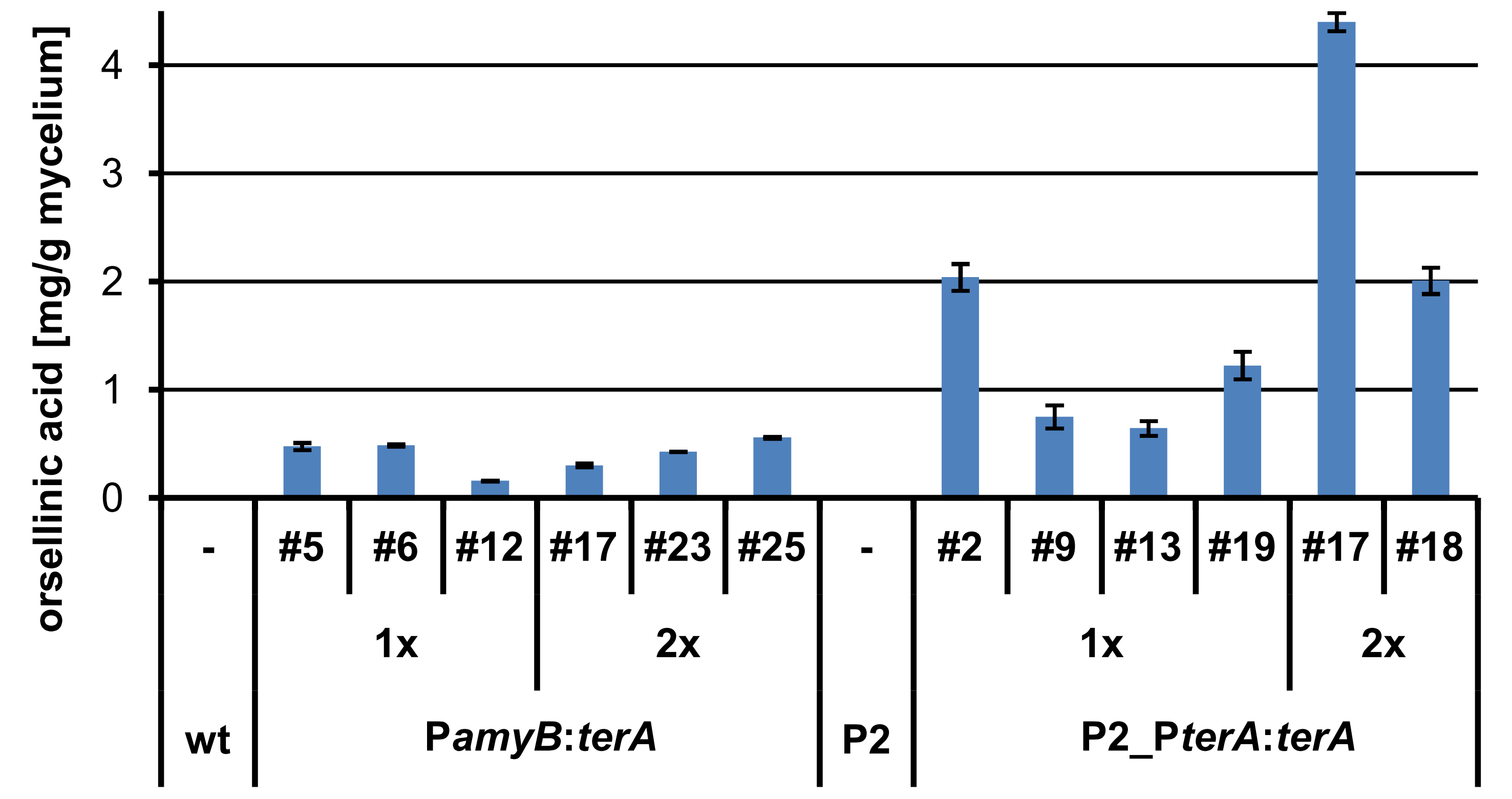


**Figure S4. Quantification of orsellinic acid.** Orsellinic acid was quantified from culture supernatants of *A. niger* strains expressing the *terA* gene under control of the *amyB* promoter (A1144_P*amyB*:*terA*) or the *terA* promoter (A1144_P2_P*terA*:*terA*)*.* The parental strains A1144 (wt) and A1144_P2 (P2) served as controls. All strains were cultivated for 48 h in AMM-G100+Gln50 liquid media. Several transformants (numbers indicated by “#”) with single (1×) and double integration (2×) were analyzed. Orsellinic acid production rates are given in milligram per gram of dried mycelium.

|  | **δ ^1^H in ppm (J/Hz)** | **δ ^13^C in ppm** |
| --- | --- | --- |
| **1** |  | 171.7 |
| **2** |  | 112.1 |
| **3** |  | 162.9 |
| **4** | 6.09, d, (2.39) | 102.6 |
| **5** |  | 159.5 |
| **6** | 6.22, d, (2.03) | 110.6 |
| **7** |  | 141.7 |
| **8** | 2.39, s | 22.4 |
| **9** |  | 167.9 |
| **10** |  | 118.8 |
| **11** |  | 158.8 |
| **12** | 6.20, d, (2.11) | 104.5 |
| **13** |  | 153.4 |
| **14** | 6.48, d, (1.78) | 113.2 |
| **15** |  | 138.1 |
| **16** | 2.25, s | 19.6 |

**Figure S5. Structure elucidation of lecanoric acid by ^1^H NMR and ^13^C NMR.** Blue arrows at the structure indicate key heteronuclear multiple bond correlation. Chemical shifts deduced from ^1^H NMR and ^13^C NMR data measured in DMSO-d6 are shown in the table.

**S3. Supplementary References**

Fleck, C.B., and Brock, M. (2010). *Aspergillus fumigatus* catalytic glucokinase and hexokinase: expression analysis and importance for germination, growth, and conidiation. *Eukaryot Cell* 9**,** 1120-1135.

Gressler, M., Zaehle, C., Scherlach, K., Hertweck, C., and Brock, M. (2011). Multifactorial induction of an orphan PKS-NRPS gene cluster in *Aspergillus terreus*. *Chem Biol* 18**,** 198-209.

Zaehle, C., Gressler, M., Shelest, E., Geib, E., Hertweck, C., and Brock, M. (2014). Terrein biosynthesis in *Aspergillus terreus* and its impact on phytotoxicity. *Chem Biol* 21**,** 719-731.
